# Highly Selective Pyrene-Anchored Halloysite Nanotube for Fluorometric Determination of 2,4,6-Trinitrophenol in Environmental and Food Samples

**DOI:** 10.1021/acsomega.4c08857

**Published:** 2025-02-18

**Authors:** Vildan Sanko, İpek Ömeroğlu, Ahmet Şenocak, Süreyya Oğuz Tümay

**Affiliations:** † Department of Chemistry, Gebze Technical University, Kocaeli 41400, Türkiye; ‡ Department of Chemistry, Hacettepe University, Ankara 06800, Türkiye; § METU MEMS Center, Ankara 06530, Türkiye; ∥ Department of Chemistry, Atatürk University, Erzurum 25240, Türkiye; ⊥ Department of Nanoscience and Nanoengineering, Atatürk University, Erzurum 25240, Türkiye

## Abstract

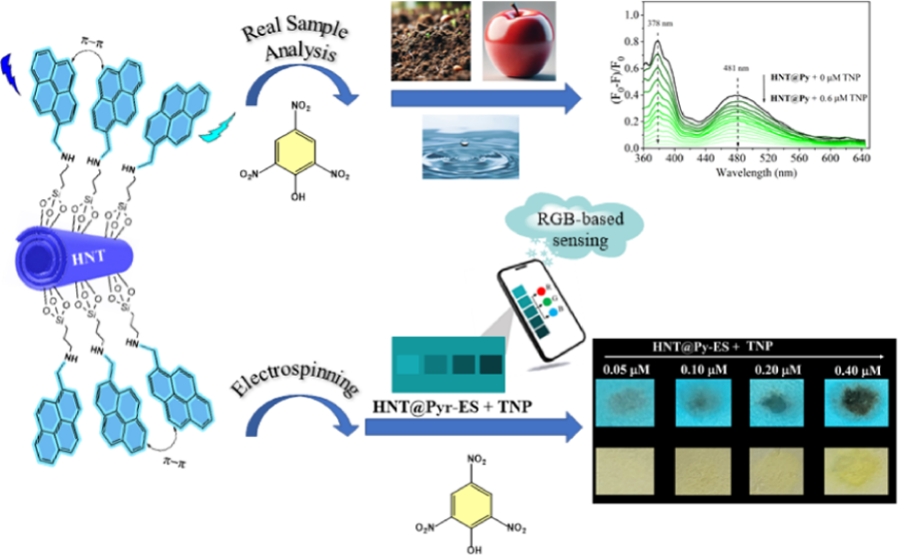

2,4,6-Trinitrophenol (TNP) is explosive, toxic, ecological, and a human health hazard and is resistant to degradation. It can cause symptoms such as headache, loss of appetite, dizziness, fever, nausea, diarrhea, and vomiting when inhaled. In this study, we aimed to develop a new halloysite nanotube anchored with pyrene moieties (**HNT@Py**) for the sensitive determination of TNP in soil, wastewater, and food samples using fully aqueous media. The **HNT@Py** was characterized structurally, morphologically, and thermally by using FTIR, UV–vis, XRD, TGA, fluorescence, SEM, and TEM analyses. The detection conditions for the assay were investigated and optimized, including competitive species, incubation time, sensor concentration, photostability, and selectivity. The limit of detection (LOD) and limit of quantification (LOQ) for TNP were found to be 14.00 nmol L^–1^ and 42.00 nmol L^–1^ in the linear response of 0.04 μmol L^–1^ and 0.60 μmol L^–1^ (*R*^2^ = 0.9962), respectively. Validation of the current method was performed using the spike/recovery test and HPLC analyses, and it was subsequently utilized to successfully detect TNP with fluorescence in soil, food, and water samples. According to the obtained results, the suggested assay is dependent on the “turn-off” emission of **HNT@Py** with the PET mechanism between electron-deficient NACs and the electron-rich pyrene moieties. The sensor was found to be easy to use, extremely sensitive, and reliable in rapidly identifying TNP in real samples, displaying excellent flexibility and resilience. Additionally, detection membranes with regular morphology were obtained by the electrospinning method using **HNT@Py** nanostructures dispersed in the PCL matrix, and RGB changes were determined via a smartphone application.

## Introduction

Nitroaromatic compounds (NACs) such as 2,4,6-trinitrophenol (TNP), 2,4-dinitrotoluene (DNT), 2,4,6-trinitrotoluene (TNT), etc., are widely used in various industries, such as pesticides, pharmaceuticals, fireworks, matches, etc.^1,2^ The fact that these compounds are resistant to degradation, pose potential explosion hazards, and exhibit toxic properties poses a danger to ecological and human health. For this reason, they are considered a global safety concern and have even been identified as “priority pollutants” for environmental remediation by the US Environmental Protection Agency.^2,3^ For this purpose, analytical studies for easy, cheap, sensitive, and selective detection of these structures remain important. The detection of NACs can be achieved by several techniques, such as mass spectrometry, optical spectroscopy, electrochemical methods, surface-enhanced Raman spectroscopy (SERS), and high-performance liquid chromatography (HPLC).^4,5^ Among these methods, fluorescence sensors are preferred for the detection of various analytes due to their sensitivity and easy applicability.^6,7^ The practical applications of fluorescence sensing systems are increasingly common and advantageous due to their solubility in a wide range of solvents, which contrasts with the limitations posed by many polymers and metal–organic frameworks (MOFs). Although “turn off-on” fluorescent sensing systems are preferred for their ability to enable visual detection, their development poses challenges. These challenges include issues related to minimal Stokes shifts and color irreversibility, which restrict their use to immediate monitoring tasks.^8,9^ Consequently, significant research efforts are directed toward the preparation of “turn on–off” π-rich nanomaterials for the detection of NACs.^10^ However, the literature reveals a scarcity of fluorescent sensors that are completely soluble/dispersible in water and effective for NAC detection.^11^

TNP (C_6_H_3_N_3_O_7_), which has a high burst rate, can cause symptoms such as headache, loss of appetite, dizziness, fever, nausea, diarrhea, and vomiting when inhaled. In addition, further exposure may cause bronchitis and pose a serious health hazard by creating carcinogenic and mutagenic effects.^12,13^ TNP can easily contaminate water, food, and soil due to its wide range of applications, high water solubility, and low degradation; for these reasons, it is classified as an environmental contaminant.^14^ Thus, developing sensitive, dependable, and effective technologies for detecting these hazardous compounds is critical to protect the environment and homeland security.^15^ In this study, fluorescence-labeled halloysite nanotubes (HNTs) were employed for the first time to detect TNP in fully aqueous media, and a composite membrane was formed with polycaprolactone (PCL) via the electrospinning method. Using the simple and low-cost electrospinning method, organic and inorganic materials can be easily combined, and products with various morphologies can be obtained.^16^ This technique, which has a wide range of applications, makes it possible to develop functional nanostructures. In terms of sensor studies, it can be said that the high surface area of the obtained nanostructured membranes causes the sensing platform to interact more with the measurement environment.^17^ Another parameter that increases the effect at this stage is the type of nanoparticle used. HNTs, known as clay with natural reserves, have a nanostructured hollow feature. While it contains alumina in its inner layer, the abundant −OH groups on its outer layer enable alternative surface modification.^18^ HNTs-based fluorescence sensors have been used to detect analytes such as the drug Imatinib,^19^ tetracycline antibiotics,^20^ and tumor cells.^21^ However, to the best of our knowledge, there has been no report in the literature on an HNT-based sensor for TNP detection by fluorescence. Thus, this study is innovative regarding the first use of HNT nanoparticles for TNP detection, new fluorophore-enabled surface modification, and electrospinning application.

Flexible membranes with high surface area obtained by the electrospinning method remarkably serve this purpose.^18^ In this study, the surface modification of HNTs with the pyrene molecule (**HNT@Py**), which has high quantum efficiency and photostability,^22^ was carried out for the first time by silanization and nucleophilic displacement reactions. The chemical structures and morphologies of the new **HNT@Py** material were elucidated by Fourier transform infrared (FTIR) spectroscopy, X-ray diffraction (XRD), thermogravimetric analysis (TGA), scanning electron microscopy (SEM), transmission electron microscopy (TEM), and ultraviolet–visible (UV–vis) methods. The optical behaviors of the new fluorescent **HNT@Py** sensors were examined by UV–vis absorption, ground state fluorescence, time-resolved fluorescence, three-dimensional fluorescence, and excitation–emission matrix analysis spectroscopies. Further experimental conditions for new fluorescent **HNT@Py** sensors, whose photophysical properties and analytical parameters were elucidated, optimized, and determined. Then, membranes with regular morphology were obtained with **HNT@Py** nanostructures dispersed into the PCL matrix using the electrospinning method, which has become accepted in the industrial market,^16^ and were used for TNP detection by the “turn-off” fluorescence method. According to the analytical data obtained, it can be concluded that the **HNT@Py**-based sensor exhibits features that can be an alternative to traditional determination methods for simple, sensitive, effective, fast, and selective fluorescence-based TNP analysis.

## Methods and Materials

### Materials and Equipment

Halloysite nanotubes, benzene, toluene, phenol, nitrobenzene (NB), nitrophenol (NP), 2,4-dinitrotoluene (2,4-DNT), 2,4,6-trinitrotoluene (2,4,6-TNT), 2,4,6-trinitrophenol (TNP), and other reagents used for experiments were purchased from Sigma-Aldrich (USA). All chemical reactions were carried out under an argon atmosphere with double-distilled water. An M615 M centrifuge (Electro-mag, Turkey) was used for the centrifugation of **HNT@Py**. The freeze-dryer was used for the drying process (Scanvac Coolsafe, Lynge, Denmark). FTIR spectra of prepared **HNT@Py** were recorded by an FTIR spectrophotometer (PerkinElmer Spectrum 100, USA). X-ray diffraction (XRD, Japan) analysis was performed by a Japanese Science Smartlab. Thermogravimetric analysis (TGA) was conducted using a Thermal Analysis System (Mettler Toledo STARe, USA). The experiment was carried out under an argon atmosphere with a flow rate of 50 mL/min, and the heating rate was set to 10 °C per minute. Scanning Electron Microscopy (FEI, Nova Nano SEM 450, USA) and Transmission Electron Microscopy (FEI, TALOS F200S TEM 200 kV, USA) were used for the analysis of surface morphological properties of **HNT@Py**. Photophysical properties and fluorescence determination measurements of both model solutions and real samples were carried out by a UV–vis electronic absorption spectrophotometer (Shimadzu 2101 UV, Japan) and a fluorescence spectrophotometer (Varian Cary Eclipse, USA). Fluorescence spectra were obtained at 25 °C while slit widths were 5 nm, and the path length of the quartz spectroscopic cuvette was 1 cm. Excitation–emission matrix analysis, time-resolved fluorescence measurements, and 3-D fluorescence were recorded by Fluorolog 3- 2iHR (Horiba-Jobin-Yvon-SPEX, France), which includes a Fluoro Hub-B Single Photon Counting Controller. The samples were excited using a 310 nm nanoLED for time-resolved fluorescence measurements (Horiba-Jobin-Yvon-SPEX, France), while a xenon lamp was employed for three-dimensional fluorescence measurements. Photon signals for time-resolved fluorescence and 3-D fluorescence measurements were obtained via the time-correlated single photon counting (TCSPC, Horiba-Jobin-Yvon-SPEX, France) module. The HPLC analyses of real samples were carried out by an Agilent HPLC system (1100 series, USA). The electrospinning process was performed using an INOVENSO NE300 electrospinner (Inovenso, Turkey) with a 3 mm rod collector.

### Photophysical Parameters

Fluorescence lifetimes of **HNT@Py** in the absence and presence of TNP were directly calculated using suitable exponential calculations, which were carried out following time-resolved fluorescence observations. **HNT@Py** and **HNT@Py** + TNP were subjected to 3-D fluorescence and excitation–emission matrix analysis (EEM) at 3 nm intervals. The **HNT@Py** and **HNT@Py** + TNP were excited between 250 and 600 nm, and the emission spectra were captured between 275 and 800 nm. The comparative method was utilized to determine the fluorescence quantum yields of **HNT@Py** and **HNT@Py** + TNP, which are significantly used for assessing photophysical behaviors. Quinine sulfate in 0.1 M H_2_SO_4_ (Φ*F*_Std_ = 0.54) was applied as the standard for both **HNT@Py** and **HNT@Py** + TNP (as described in eq 1).^23^
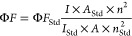
1

In the provided equation, the parameters for the standard’s quantum yield (Φ*F*_Std_ = 0.54), the refractive indices, absorbance, and band areas of fluorescence are denoted by Φ*F*, *n*, *A*, and *I*, respectively. To mitigate agglomeration and minimize the inner filter effect, all optical investigations were carried out in highly diluted dispersions. Other essential photophysical parameters relevant to fluorescence detection mechanisms and sensing, including nonradiative (*k*_nr_), radiative (*k*_r_) rate constants, and average lifetime values (τ_a_) were calculated using eqs 2-4.^24^
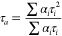
2
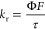
3
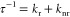
4

### Synthesis of Pyrene-Anchored Halloysite Nanotube (HNT@Py)

A silane-modified halloysite nanotube (HNT@APTES) made of (3-aminopropyl)triethoxysilane was produced and purified according to the literature.^25^ Then, 30 min of ultrasonication were used to disperse 0.50 g of HNT@APTES in 10 mL of acetonitrile (ACN). 1.50 g (10.85 mmol) of K_2_CO_3_ was introduced to the resultant mixture in the presence of an inert atmosphere and stirred for 30 min at a temperature of 25 °C. Then, 1.00 g (3.39 mmol) of 1-(bromomethyl)pyrene was added to this mixture, and the reaction was performed at 25 °C for 72 h. Upon completion of the modification, the crude product was collected via centrifugation at 4000 rpm for 10 min and subsequently washed three times with dichloromethane, THF, ethanol, and acetone. Then, **HNT@Py** was dried in a vacuum oven at 50 °C, and a yellowish solid was obtained.

### Real Samples and Fluorometric Determination Assay of TNP via HNT@Py

All samples were collected from Kocaeli in Turkey and stored at 4 °C in the refrigerator. Soil and food samples were purchased from a local market and agricultural land in Kocaeli, Turkey, and large particles of soil samples were separated by a sieve. After the soil sample was dried for 24 h at 110 °C, the acetone and ethanol-based extraction was applied to retrieve TNP from soil and food samples following the previous reports with some changes.^26^ Briefly, 5.0 g of dried soil and food samples were treated with 50 mL of 1:1 (v/v) acetone and ethanol and homogenized for 1 h with an ultrasonic bath, separately. Then, a 0.22 μm syringe filter was used for the obtained extract solution, which was stored at −20 °C in the dark prior to use. The stored samples were directly used in the developed a fluorimetric determination method, which depended on the highly sensitive, fast, and selective interaction of **HNT@Py** with TNP.

0.5 mg mL^–1^**HNT@Py** was prepared in water as a stock dispersion. Stock solutions of metal ions (nitrate salts) and anions (sodium salts) were prepared in double-distilled water. In addition, stock solutions of nitroaromatic compounds (benzene, toluene, phenol, NB, NP, 2,4-DNT, 2,4,6-TNT, and TNP) and single molecules (ascorbic acid, glucose, gallic acid, and fructose) were prepared in ethanol. The metal mixture for selectivity studies was prepared with Ag^+^, Al^3+^, Ba^2+^, Cr^3+^, Co^2+^, Cu^2+^, Cs^+^, Ca^2+^, Cd^2+^, Fe^2+^, Fe^3+^, Hg^2+^, K^+^, Li^+^, Mg^2+^, Mn^2+^, Na^+^, Ni^2+^, Pb^2+^, and Zn^2+^ and the corresponding nitrate salts were used with this mixture. Fluorescence measurement of TNP in real samples and optimization studies were recorded using a relative fluorescence signal change of **HNT@Py** at the monomer vs excimer emission intensity ratio (*I*_378_/*I*_481_). A 0.80 mL portion of **HNT@Py** was taken from the stock dispersion and added to a 2.00 mL volumetric flask. Subsequently, 0.40 mL of the real sample was introduced into the volumetric flask, and using double-deionized water, the final volume was completed to 2.00 mL. Prior to conducting fluorescence measurements, the resulting solution was agitated vigorously for 10 s. This quenching was proportional to the increasing amount of TNP up to 0.60 μM which was appropriate for determining the TNP concentration of real samples. Spike/recovery tests and the conventional HPLC method were performed on real samples under ideal conditions to determine the accuracy of the provided method. For HPLC analysis, the C18 column (5 nm, 250 × 4.6 mm^2^ i.d., SVEA, Nanologica, Sweden) was used as a stationary phase, and 357 nm was chosen as the detection wavelength with a 3 nm slit width. Furthermore, a methanol:water (90:10) mobile phase was employed, with a flow rate of 1.400 mL·min^–1^, and isocratic elution was utilized at room temperature. For HPLC analysis, the sample injection volume was 10 μL, and the TNP retention period was 1.80 min. The stock solution (2000 μM) of TNP was prepared in the mobile phase, and various TNP concentrations between 1000.00 μM and 25.00 μM were applied to obtain the calibration curve of HPLC analysis.

### Production of HNT@Py-Doped Electrospun Nanofibers for RGB-Based TNP Detection

A PCL solution containing modified and unmodified HNT was prepared for use in the electrospinning study designed in the current study. 5% w/v PCL was mixed in DMF:DCM (1:1 v/v) at room temperature using a magnetic stirrer for approximately 2 h. A solution containing only PCL was used as the negative control group. As a positive control group, a 30% w/v unmodified HNT dispersion in PCL solution was prepared using a sonicator for approximately 1 h. Sensor working groups containing 30% (w/v) **HNT-Py** dispersed in PCL were utilized. The prepared groups were placed into a 10 mL syringe and an 18-gauge stainless steel needle at room temperature. The flow rate was 0.75 mL/hour, the distance between the aluminum foil as a collector and the syringe was 12 cm, and the voltage was 25 kV.^27^

## Results and Discussion

### Synthesis and Characterization of HNT@Py

**Scheme 1:**
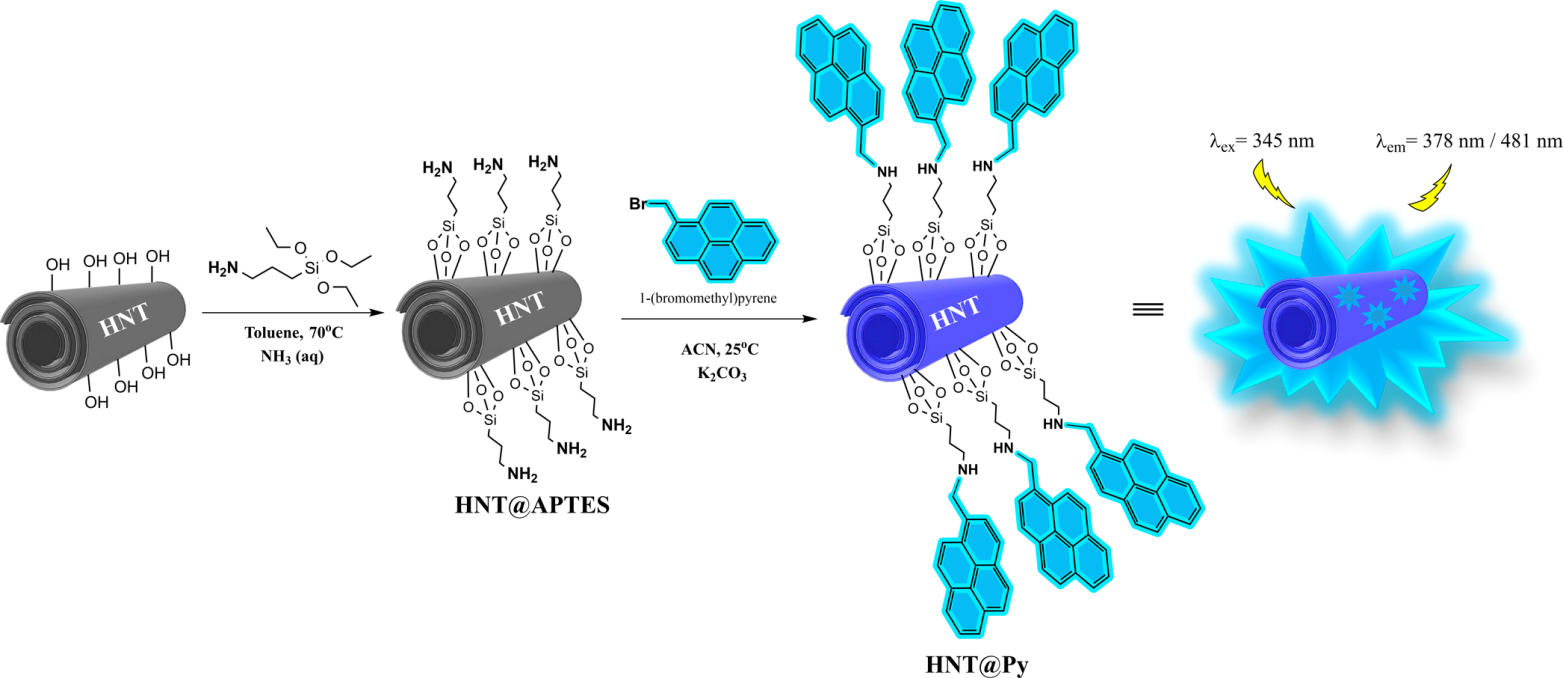
Surface Modification of HNTs to Obtain Pyrene-Anchored Fluorescent **HNT@Py**

There are many reports in the literature about the production and application of TNP fluorescence sensors based on functional nanomaterials.^28,29^ However, there are no HNT-based sensor studies for TNP detection by the fluorescence method in the literature. HNTs, natural aluminosilicate clay minerals, have become promising materials thanks to their desirable properties, such as hydrophilicity, nontoxicity, biocompatibility, nondegradation, and low cost.^30^ Because of these advantages, they are used in different applications such as drug delivery, adsorption, catalysis, electrochemical sensors, and fluorescence probes.^31^ HNTs can be functionalized by surface and structural modifications to increase their solubility and bioavailability because they are easily affected by hydrogen bonds and cause unstable solubility.^32^ Siloxane (Si–O–Si) and aluminol (Al–OH) make up most of the interior and exterior surfaces of HNTs, allowing for selective surface functionalization.^33^ For this reason, in an innovative approach, the surface of HNTs was modified with pyrene molecules by nucleophilic displacement reactions to execute sensitive and selective “turn-off” signal changes after the addition of TNP and real sample analyses (Scheme 1). The pyrene molecule was chosen as the fluorophore because of its widely recognized photophysical features, and pyrene derivatives exhibited fluorescence emission from both monomers and excimers.^34^ The surface functionalization of HNTs was performed using APTES.^25^ Subsequently, **HNT@Py**, which are HNTs modified with pyrene, was synthesized by replacing the primary amine-functionalized HNTs with 1-(bromomethyl)pyrene through a nucleophilic displacement reaction in acetonitrile for 24 h. The **HNT@Py** designed for utilization in the sensor platform was precipitated by centrifugation and purified by washing three times each with dichloromethane, ethanol, water, and acetone. FTIR, XRD, TGA, SEM, TEM, UV–vis, and fluorescence techniques were used to examine the morphological, thermal, and structural characteristics of the pyrene-anchored HNTs (**HNT@Py**) following purification.

In the FTIR spectrum of HNTs (Figure S1a), O–H stretching of inner surface hydroxyl groups, attributed to Al–OH stretching vibrations, were observed at 3694 and 3622 cm^–1^. The inner Si–O stretching vibration and O–H deformation vibration of the inner hydroxyl groups were observed at 1006 and 909 cm^–1^.^35^ After surface modification with APTES, the stretching and bending vibrations of the −CH_2_ groups were observed at 2935 and 1325 cm^–1^, respectively.^36^ The pyrene structure exhibited aromatic C–H vibrations at 3038 cm^–1^, which resulted from the nucleophilic displacement reaction of primary amine-functionalized HNTs with 1-(bromomethyl)pyrene. The XRD patterns in Figure S1b illustrate the crystalline structure of HNTs, HNT@APTES, and **HNT@Py**. The XRD chart shows recognizable and sharp peaks between 2θ = 11.86–12.34, correlated with the plane of 001 in the nanotubes. Additionally, there are peaks at 2θ = 19.94–20.01 and 2θ = 26.62–26.72, which can be attributed to the 110 and 002 planes, respectively.^36,37^ The modification of HNTs did not alter the distance between the planes, suggesting that APTES and pyrene are situated on the exterior surface rather than between the layers. The XRD patterns demonstrate an unaltered structure of the HNTs, indicating that the crystalline structure remained unchanged or undamaged throughout the functionalization processes.^38^

Thermal properties of **HNT@Py** were investigated in comparison with HNTs and HNT-APTES (Figure S1c). From the TGA thermogram, 5% and 10% mass losses between 50 and 150 °C were observed for HNTs and HNT@APTES, respectively. The losses in weight were due to the evaporation of absorbed water from the outer and inner channels of both HNTs and HNT@APTES. Additionally, the decrease in mass of 16.63% for pristine HNT between 300 and 700 °C corresponded to the dehydroxylation of hydroxyl groups from the remaining Al–OH and Si–O–Si structures on the surface of HNTs, and 20.10% for HNT@APTES belonging to the decomposition of aminosilane attached to the surface of HNTs.^39^ In addition, the mass losses of 25.63% at 700 °C were observed for **HNT@Py**, which demonstrated that surface modification of **HNT@Py** was successfully achieved.^40^ UV–vis absorption spectra of 1-(bromomethyl)pyrene, HNT@APTES, and **HNT@Py** in ethanol were displayed in Figure S1d. Although the UV–vis absorption of HNT@APTES did not show any noticeable absorption peaks between 200 and 600 nm, **HNT@Py** exhibited absorption peaks at 231–242 nm, 265–275 nm, and 311–344 nm, which are in good agreement with the absorption bands of labeled pyrene moieties, which generally exhibit four absorption bands at nearly 240, 270, 330, and 370 nm, and they are assigned to the S_4_ ← S_0_, S_3_ ← S_0_, S_2_ ← S_0_, and S_1_ ← S_0_ transitions, respectively.^41^ The most crucial evidence for modification of the HNT surface with the pyrene moieties is the fluorescence spectra of the sensing platform (Figure S1e). As can be seen from Figure S1e, **HNT@Py** demonstrated both excimer and monomeric fluorescence of the pyrene moieties that centered at 378 and 481 nm, respectively.^42^ The formation of excimer emission as a result of the π–π interaction of the pyrene moieties on the surface of the **HNT@Py** suggests that modification has successfully occurred.^43^

**Figure 1:**
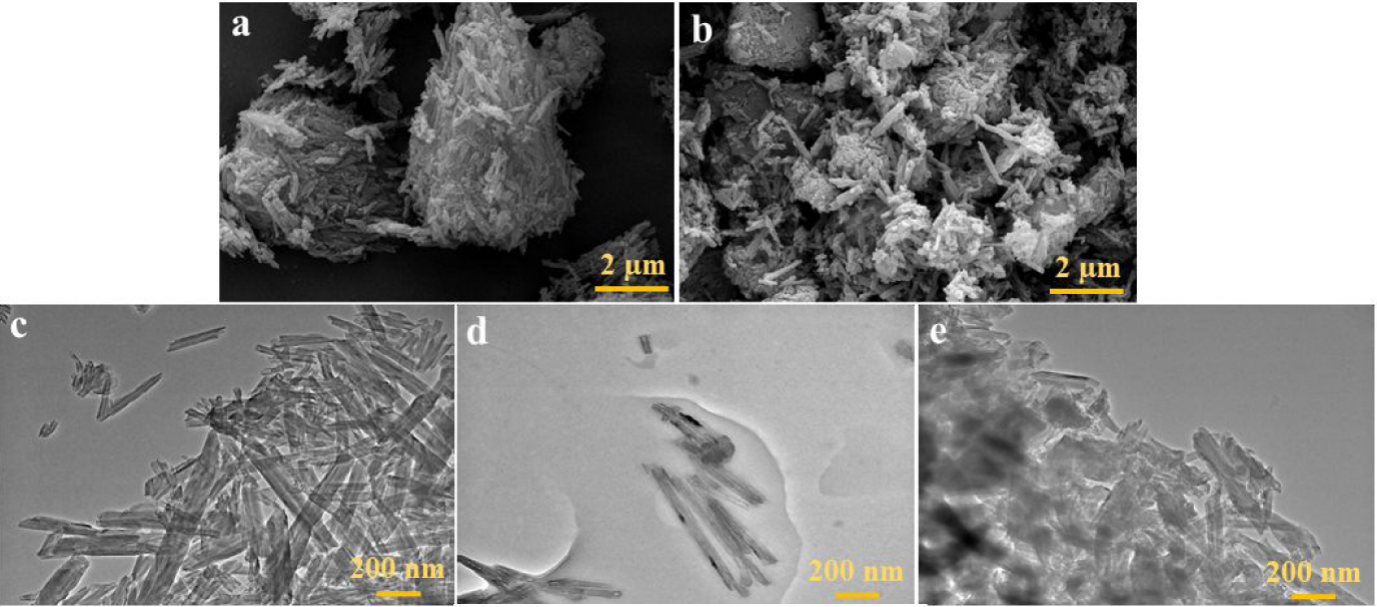
SEM images of (a) HNT, (b) **HNT@Py**, and TEM images of (c) HNT, (d) HNT@APTES, and (e) **HNT@Py**.

SEM and TEM were used to examine the surface morphology of the novel **HNT@Py** sensor system. Figure 1a,b displays the nanotubular structures with an open-ended lumen in the SEM images of pristine HNTs and **HNT@Py**, respectively.^44^ Also, according to the analysis of TEM analyses with the ImageJ program, **HNT@Py** has a more rugged surface and a larger outer diameter (111.09 ± 18.50 nm) compared to pristine HNTs (92.21 ± 14.17 nm). In addition, according to TEM images, it was determined that pristine HNTs significantly retained their characteristic length and tubular structure after surface modification (Figure 1c–e).^45^Figure 1c represents the natural structure of HNT with a smooth surface and walls; Figure 1e shows the structural change that occurs due to the modification of HNT in an alkali environment, with irregularity on the surface and partially damaged edges. Based on the obtained morphological images, a slight abrasion occurred on the HNT surface due to the pyrene modification in an alkali environment.^46^

### Photophysical Behaviors of HNT@Py

The photophysical behaviors of **HNT@Py** were evaluated by time-resolved fluorescence, steady-state fluorescence, and UV–vis spectroscopies. The absorption properties of the **HNT@Py** were investigated in various solvents at the same concentration (0.5 mg·mL^–1^). The absorption bands between 255 and 281 nm and 326–351 nm of **HNT@Py** are shown in Figure S2a. Since HNTs do not show a significant absorption peak in the scanning scope,^37^ the UV–vis region displays bands that arise from the π–π* transition of the pyrene moieties.^47^ The absorption characteristics of the **HNT@Py** sensor remained consistent with the differentiation of solvents, except for the absorbance. The varying dispersion properties of the **HNT@Py** sensor in different solvents may have caused the absorbance differences. Also, absorption spectra were recorded at concentrations that ranged from 0.5 mg mL^–1^ to 0.12 mg mL^–1^ in different solvents to determine the concentration impact on the electronic properties of **HNT@Py** (Figure S3). When the concentration of **HNT@Py** decreased, the absorbance values declined correspondingly without shifting the absorption wavelength (Figure S3).

The identical analytical parameters of absorption spectra were applied for the evaluation of fluorescence behaviors of **HNT@Py** when excited at 345 nm (Figure S2b). The pyrene compound can exist in solution as an excimer or monomer, and excimers differ from monomer pyrene compounds in their spectroscopic characteristics. These attributes include a redshift in emission wavelength and changed fluorescence lifetimes. Environmental factors such as temperature, viscosity, pressure, pH, and molecular confinement all impact the excimer production process.^48^ To investigate the coexistence of monomer and excimer, the fluorescence emission spectra of **HNT@Py** in different solvents were obtained, and **HNT@Py** showed an obvious coexistence in the studied solvents. Furthermore, the data suggest that no clear correlation exists between solvent polarity and excimer intensity. In the aqueous dispersion, the intensity of the fluorescence band corresponding to monomer emission exceeds that of the excimer emission. This observation can be attributed to the favorable solvent characteristics of water concerning **HNT@Py**.^49^ Also, this result indicated that the pyrene excimers in **HNT@Py** were mainly generated by the approach of two or more pyrene monomers. **HNT@Py** showed monomer emission wavelengths of 364–396 nm and excimer emission wavelengths of 474–481 nm in the studied solvents. Also, the monomer vs excimer emission intensity ratio (*I*_378_*/I*_481_) of the **HNT@Py** in water was calculated as ∼2.0, and this ratio remained the same with increasing or decreasing concentration. The **HNT@Py** demonstrated a Stokes shift of 134 nm in the water suspension (Figure S2c). In Figure S4, it can be observed that the emission region (364–396 nm and 474–481 nm) of the **HNT@Py** remained unaffected by alterations in the solvent system. Moreover, the emission intensity decreased in direct proportion to the dilution with no accompanying wavelength shift, indicating that self-quenching is not present in the solutions. Photophysical parameters, including the absorption coefficient (ε), fluorescence quantum yield (Φ*F*), and fluorescence lifetime (τ_0_) of **HNT@Py** in water, are summarized in Table S1.

### Optimization of Sensing Conditions for TNP Determination

TNP contaminates soil and groundwater because of its high water solubility and may cause eye/skin irritation, unconsciousness, weakness, muscle pain, and kidney problems.^50^ To detect TNP, it is essential to investigate novel fluorescent materials that are both highly selective and sensitive.^51^ In this study, thorough research was conducted to examine the experimental factors that may impact the fluorescence “turn-off” determination of TNP in water, food, and soil samples using pyrene-anchored halloysite nanotubes (**HNT@Py**). The investigation involved evaluating and optimizing experimental parameters, including selectivity, competitive species, sensor concentration, and incubation time.

### Selectivity of HNT@Py and Effect of Competitive Species

**Figure 2:**
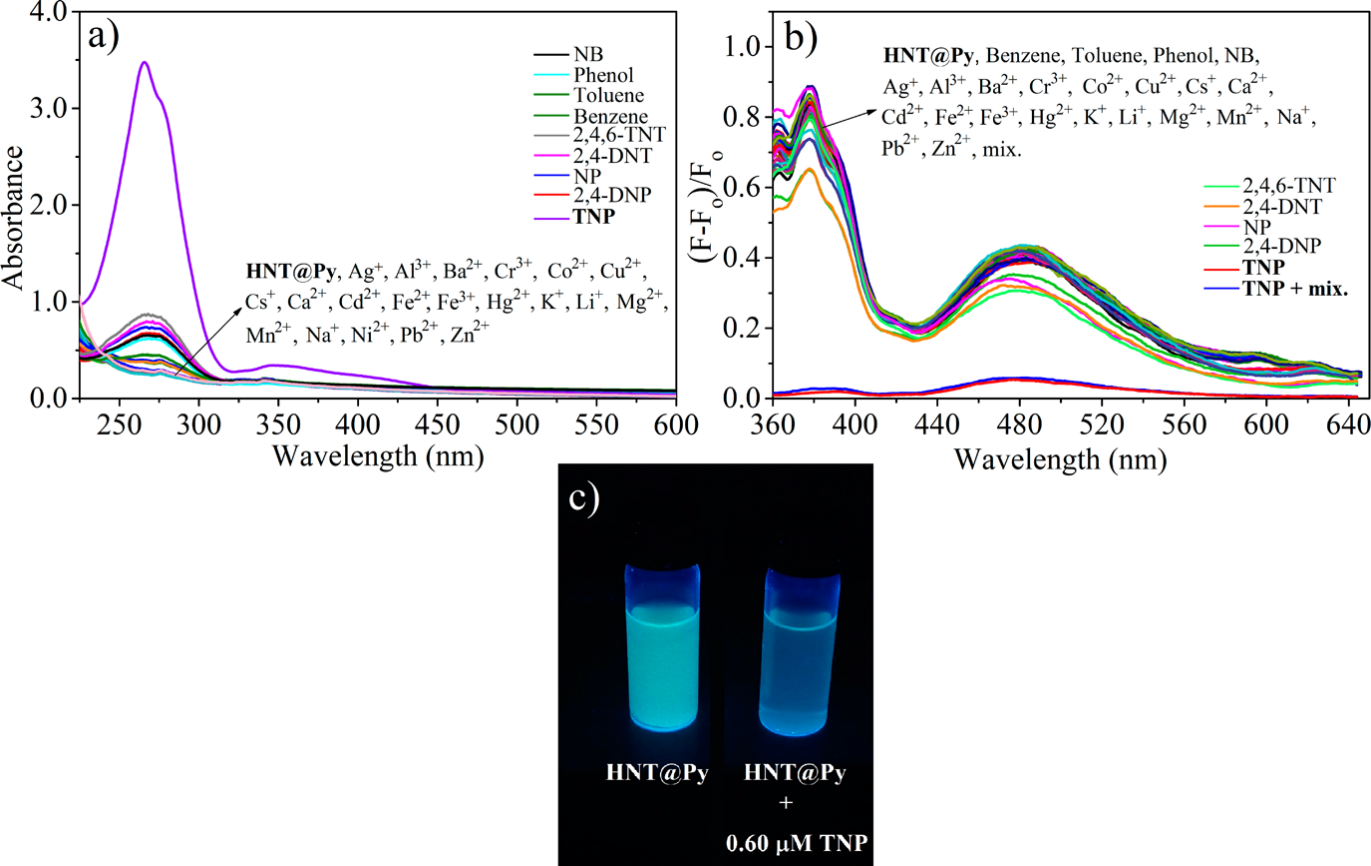
(a) UV–vis absorption, (b) fluorescence signal (λ_ex_= 345 nm), and (c) naked-eye color variation (λ_ex_= 365 nm) for **HNT@Py** after interaction with various NACs and metal ions in water.

It is crucial to have a high level of selectivity for fluorescence signal-based analysis in real samples to advance the development of cutting-edge fluorescence sensors. Therefore, the selectivity was confirmed by measuring UV–vis and fluorescence in water at 25 °C. Initially, ultrasonication was employed for 10 min to create a water dispersion of **HNT@Py** (0.5 mg·mL^–1^). Then, the UV–vis absorption and fluorescence emission responses of the **HNT@Py** were evaluated after treatment with 0.60 μM of nitroaromatic compounds (benzene, toluene, phenol, NB, NP, 2,4-DNT, 2,4,6-TNT, and TNP) and 0.60 μM of various tri-, di-, and monovalent cations (Ag^+^, Al^3+^, Ba^2+^, Cr^3+^, Co^2+^, Cu^2+^, Cs^+^, Ca^2+^, Cd^2+^, Fe^2+^, Fe^3+^, Hg^2+^, K^+^, Li^+^, Mg^2+^, Mn^2+^, Na^+^, Ni^2+^, Pb^2+^, and Zn^2+^) were included in the water dispersion of **HNT@Py** (Figure 2a,b). Based on the data in Figure 2a, the absorption peaks of **HNT@Py** did not effectively change upon the introduction of benzene, toluene, phenol, NB, NP, 2,4-DNT, and 2,4,6-TNT, and no spectral changes were observed with the addition of other competitive species such as metal ions. Above all, the addition of TNP to the water dispersion of the **HNT@Py** caused a significant change in absorption bands at 266 nm (∼13.5-fold) and 350 nm (∼2-fold) enhancement. The absorption bands of the **HNT@Py** changed significantly after the addition of TNP. In contrast, other nitroaromatic compounds (benzene, toluene, phenol, NB, NP, 2,4-DNT, 2,4,6-TNT) caused a negligible change in the absorption bands.^52^ The rearrangement in the electronic structure of **HNT@Py** and the shift in UV–vis absorption spectra upon the addition of TNP might be attributed to the efficient transfer of electrons between the electron-rich pyrene moieties and the electron-deficient NACs.^9^

The selective fluorescence response of the **HNT@Py** toward TNP is one of the most crucial parameters for assessing the fluorescence of TNP in water, food, and soil samples. Consequently, the selectivity of the presented sensor system for TNP was assessed using fluorescence emission spectroscopy, conducted under identical experimental conditions with absorption studies. The intensity of **HNT@Py** was unaffected by testing other nitroaromatic compounds and metal ions. At the same time, **HNT@Py** exhibited complete fluorescence quenching at monomer emission (378 nm) and excimer emission (481 nm) after adding the TNP solution (Figure 2b). The monomer emission to excimer fluorescence ratio (*I*_378_/*I*_481_) was determined to provide a better understanding of the selectivity. The relative fluorescence change of the monomer/excimer emission ratio for **HNT@Py** was 77.35% after treatment with TNP, indicating that the fluorescence of **HNT@Py** had been significantly quenched with the addition of TNP. The generation of a donor–acceptor complex by electron transfer after adding TNP resulted in the quenching of monomer/excimer fluorescence.^53^ The excited electron of the pyrene group deactivates over the energy level of the analyte when the electron-rich pyrene moieties contact the electron-deficient NACs. This state is known as fluorescent quenching or fluorescence “turn-off”, and it varies depending on the concentration of the analyte.^54^

The water dispersion of **HNT@Py** underwent a color change visible to the naked eye when it was excited with a fluorescent light source following the addition of TNP. The water dispersion of the **HNT@Py** was bright blue/green before interaction with TNP; still, it became colorless after that, as shown in Figure 2c.

In contrast, the dispersion colors were unaffected by the other competing nitroaromatic compounds and metal ions. The results indicated that **HNT@Py** is suitable for the rapid, straightforward, and sensitive detection of TNP in real samples, exhibiting a highly selective “turn-off” signal change toward TNP. The impact of competing species on the relative fluorescence response of **HNT@Py** was examined in water for 3.00 μM of different nitroaromatic compounds (benzene, toluene, phenol, NB, NP, 2,4-DNT, 2,4,6-TNT, and TNP), single molecules (ascorbic acid, glucose, gallic acid, and fructose), anions (I^–^, HSO_4_^–^, Cr_2_O_7_^2–^, H_2_PO_4_^–^, CO_3_^2–^, CN^–^, HCO_3_^–^, Br^–^, NO_3_^–^, NO_2_^–^, CrO_4_^2–^, S_2_O_3_^2–^, F^–^, CH_3_COO^–^, Cl^–^, S^2–^, SO_4_^2–^, and SCN^–^) and cations (Ag^+^, Al^3+^, Ba^2+^, Cr^3+^, Co^2+^, Cu^2+^, Cs^+^, Ca^2+^, Cd^2+^, Fe^2+^, Fe^3+^, Hg^2+^, K^+^, Li^+^, Mg^2+^, Mn^2+^, Na^+^, Ni^2+^, Pb^2+^, and Zn^2+^). As illustrated in Figure S5, the relative fluorescence response of **HNT@Py** remained unaffected by competing species, thus affirming its high selectivity for TNP. The fluorescence response of the pyrene moieties within the halloysite nanotubes, measured by the *I*_378_/*I*_481_ ratio, was found to be contingent upon the presence of TNP. This dependence is attributed to the combined impact of electron transfer processes between the fluorescent sensor and TNP. As a result, it can be inferred that the fluorimetric determination of trace concentrations of TNP can be reliably conducted using **HNT@Py** in water dispersion, even in the presence of other competing substances. The reusability and the effect of tested interfering species on the relative fluorescence signal change of **HNT@Py** + TNP were evaluated by the same competitive species. As can be seen from Figure S5b, further treatment of interfering species with **HNT@Py** + TNP demonstrated a negligible effect on the analytical signal, which pointed out the high selectivity of **HNT@Py**. The reusability of TNP sensors has been examined with TEA and urea derivatives in the literature.^55,56^ However, tested species, including urea, thiourea, TEA, cations, single molecules, and anions, did not cause remarkable recovery of the signal of **HNT@Py**.

### Concentration Effect of HNT@Py

The fluorescence intensity is directly influenced by the concentration of fluorescent sensors.^57^ Consequently, when all other conditions remained unaltered, 0.60 μM TNP was introduced to varying concentrations of **HNT@Py** (ranging from 0.1 mg mL^–1^ to 1.0 mg mL^–1^) in a water dispersion. In Figure S6a, it can be observed that the relative fluorescence signal of **HNT@Py** + TNP peaked when the concentration of **HNT@Py** was raised from 0.10 mg mL^–1^ to 0.5 mg mL^–1^ but decreased beyond 0.5 mg mL^–1^. The observed reduction in fluorescence intensity beyond this concentration may be attributed to intermolecular self-quenching among the pyrene groups or the agglomeration of HNTs.^58,59^ In light of these findings, the starting **HNT@Py** concentration for the remaining assays was set at 0.5 mg mL^–1^ to avoid HNT agglomeration or self-quenching and enable the determination of TNP in soil, food, and water samples.

### Effect of Time on Measurement

To achieve consistent and repeatable analytical results for the “turn-off” fluorescence detection of TNP in real samples, the interaction time between the sensor and the analyte is an important parameter. This was accomplished by optimizing the water dispersion of **HNT@Py** (0.5 mg·mL^–1^) and 0.60 μM TNP at various times to find the impact of interaction time on the emission response of **HNT@Py** + TNP under constant conditions. The relative signal change of **HNT@Py** + TNP is almost constant between 10 and 20 s, as shown in Figure S6b. To get a consistent and reproducible analytical signal for the “turn-off” fluorescence assessment of TNP in real samples, 10 s was used as the interaction period.

### Photostability of HNT@Py and HNT@Py + TNP

The photostability of the fluorescent sensor and its products is influenced by the interaction of the sensor with the analyte, which is a critical factor in determining fluorescence intensity throughout the process. Furthermore, nanomaterial-based fluorescent sensors must exhibit high photostability to enable real-time monitoring of dynamic events such as the rapid detection of TNP in real samples. Hence, the photostability of **HNT@Py** (0.5 mg mL^–1^) and **HNT@Py** + TNP (0.5 mg mL^–1^**HNT@Py** + 0.60 μM TNP) was assessed under optimized conditions for 60 min in daylight. As seen in Figure S6c, the relative fluorescence response of **HNT@Py** and **HNT@Py** + TNP remained nearly constant throughout the 60 min. Therefore, it can be concluded that both **HNT@Py** and **HNT@Py** + TNP demonstrated high photostability for the “turn-off” fluorescence detection of TNP in real samples.

### Effect of pH on Measurements

The pH of the sensing medium can affect both the stability and fluorescence signal change of the nanosensing system. Therefore, the effect of pH on the sensing medium was evaluated with a Britton-Robinson (BR) buffer system in the range of 2–12. As can be seen from Figure S6d both **HNT@Py** and **HNT@Py** + TNP show stable relative fluorescence change between pH 4.0–9.0 with negligible effect. These results demonstrate that the stability and fluorescence response of **HNT@Py** and **HNT@Py** + TNP were consistent over a wide range of pH changes. This range allows **HNT@Py** to be suitable for real sample analysis in targeted sensing media, including water, soil, and food samples.

### Fluorescence Sensing Mechanism for TNP Detection

In the photoinduced electron transfer mechanism (PET), one of the fluorescence quenching mechanisms of TNP, an electronic transition occurs in the fluorophore after illumination, leading to an excited state. After the donor–acceptor interaction between the electron-rich fluorophore and the electron-deficient TNP, the excited fluorophore transfers an electron from its LUMO level to the LUMO level of the receptor. This transfer leads to quenching, as the resulting complex subsequently returns to the ground state in a nonluminescent form.^60,61^ Since pyrene and its derivatives have a planar aromatic surface that tends to form strong electron transfer complexes with electron-deficient analytes and unique photophysical characteristics, they are widely used as fluorescent probes in various applications.^62^ The π–π interaction between the pyrene moieties and the NACs, performing as a quencher, is responsible for the efficient quenching of the fluorescence of the NACs to the electron-rich compounds. It is well known that the π-conjugated planar structure facilitates the π–π interaction between pyrene and NACs.^63^ Thus, the fluorescence quenching observed in the HNTs-based fluorescent sensor, modified with pyrene moieties as the fluorophore through a nucleophilic displacement reaction, can be attributed to photoinduced electron transfer (PET) processes from pyrene to TNP. Upon the addition of the TNP solution, **HNT@Py** exhibited complete fluorescence quenching at both monomer emission (378 nm) and excimer emission (481 nm). Furthermore, the pyrene moieties may be responsible for the free intense bright blue emission of **HNT@Py**. Pyrene-based fluorophores containing multiple pyrene moieties can demonstrate either monomer or excimer emission via π–π and CH-π interactions. The resultant changes in signal can be effectively employed for the quantitative detection of target species in real samples.^64^ Time-resolved fluorescence measurements of **HNT@Py** and the **HNT@Py** + TNP complex were conducted to investigate the fluorescence signal change. The lifetimes for **HNT@Py** in the absence and presence of TNP were derived through triexponential fitting of their fluorescence decay profiles. The average fluorescence lifetimes were found to be 25.48 ns for **HNT@Py** and 24.80 ns for **HNT@Py** + TNP, as summarized in Table 1. Fluorophores’ quenching through fluorescence can occur through two processes known as static and dynamic quenching. While dynamic quenching occurs through a diffusion-controlled mechanism in the excited state, static quenching depends on a nonluminescent ground state complex formation.^65^ Consequently, information on the fluorescence quenching process may be obtained from the relationship between the change in fluorescence response and an increase in quencher concentration. Time-resolved fluorescence measurements can provide further insights into the fluorescence mechanism, as the fluorescence lifetime of the free sensor (τ_1_/τ_0_ ≈ 1) remains unaffected by the formation of the nonfluorescent ground state complex associated with static quenching. However, it significantly changes during the dynamic quenching mechanism. The Stern–Volmer graph was utilized to approve the quenching process. Static quenching was shown by a linear graph from the plotting of *I*_0_/*I* versus [TNP] with a *y*-axis intercept of 1.0, whereas dynamic quenching was indicated by a positive deviation from the same graph.^66^ Upon the addition of TNP, the Stern–Volmer plot yielded a linear graph with a *y*-axis intercept of 1.0, indicating that static quenching effectively diminishes the fluorescence signal of **HNT@Py** (Figure S7a). However, after 4.0 μM of TNP, the positive deviation was observed, which indicated the contribution of the dynamic quenching process after treatment with the higher concentration of quencher.^67,68^

**Table 1:** Fluorescence Quantum Yields and Time-Resolved Fluorescence Parameters of **HNT@Py** in the Absence and Presence of TNP

	τ_1_ (ns)	τ_2_ (ns)	τ_3_ (ns)	α_1_	α_2_	α_3_	τ_a_ (ns)	Φ*F*	*k*_r_ (×10^6^)	*k*_nr_ (×10^6^)
**HNT@Py**	6.961	1.342	31.524	0.3832	0.3052	0.3116	25.48	0.20	7.85	31.39
**HNT@Py** + TNP	7.068	30.945	1.475	0.361	0.2998	0.3393	24.80	0.02	0.80	39.50

To investigate the fluorescence quenching process, time-resolved fluorescence spectroscopy was employed for lifetime studies (Figure S7b). Time-resolved fluorescence measurements for **HNT@Py** (τ_0_) and **HNT@Py** + TNP (τ_1_) were carried out to obtain more precise information concerning the quenching mechanism. The ratio of the fluorescence lifetimes (τ_1_/τ_0_ ∼0.97) indicates that there was very little change in the fluorescence lifetime of **HNT@Py** when the analyte was added to the fluorescence sensor system. The ratio of fluorescence lifetimes of a sensor in the absence (τ_0_) and presence (τ_1_) was found to be around 1.0, indicating that the production of nonfluorescent complexes were the main source of the fluorescence quenching response of **HNT@Py**, according to the experimental data. Furthermore, the fluorescence quantum yields were 0.20 and 0.02 for **HNT@Py** and **HNT@Py** + TNP, respectively, indicating a significant decline following the interaction with TNP (Table 1).

The parameters obtained from time-resolved measurements, including the radiative (*k*_r_) and nonradiative (*k*_nr_) rate constants, were assessed to gain deeper insights into the sensing mechanism. The quantum yields of **HNT@Py** were measured both in the absence and presence of TNP, yielding 0.02 and 0.20, respectively. Notably, a decrease in fluorescence quantum yield was observed with the addition of TNP, which can be attributed to effective charge transfer from the electron-rich **HNT@Py** to the electron-deficient TNP.^69^ The nonradiative (*k*_nr_) and radiative rate constants (*k*_r_) for both **HNT@Py** and the **HNT@Py** + TNP complex were determined using eqs 3 and 4. It was found that the radiative rate constant of **HNT@Py** significantly decreased after interaction with TNP. Conversely, the nonradiative rate constants increased after treatment.^70^ This significant decline in the radiative rate constant and fluorescence quantum yield, alongside the increase in the nonradiative rate constant while average fluorescence lifetimes remained relatively unchanged, can be attributed to the formation of a nonradiative ground-state complex between **HNT@Py** and TNP. This complex results in a “turn on–off” fluorescence signal change due to an inhibition in radiative pathways.^9,71^

In order to evaluate nonradiative ground state complex formation, UV–vis absorption spectra of TNP were recorded in water and depicted in Figure S7c. As can be seen from the UV–vis spectra, the absorption bands of TNP, which have a maximum at 334 nm, significantly overlap with the emission of **HNT@Py**, indicating a possibility of an inner filter effect (IFE) for fluorescence quenching when considering the nearly unchanged fluorescence lifetime (τ_1_/τ_0_ ∼0.97) that pointed to static quenching.^72^ Therefore, it can be concluded that PET is not the sole mechanism for the fluorescence quenching response, and the IFE mechanism may also contribute to the quenching effect after interaction with TNP.^73^ The particle-size and solid-state fluorescence responses of **HNT@Py** in the presence and absence of TNP were also examined (Figure S8). The particle sizes of the HNT, **HNT@Py**, and **HNT@Py** + TNP were determined as 2250 ± 195.22 nm, 1155 ± 82.30 nm, and 1036 ± 78.40 nm, respectively, by DLS analysis in water dispersions. After the HNT nanoparticle was modified with Py, the decrease in particle size was consistent with the morphological images and slight abrasion in an alkali environment.^46^ The change in the particle distribution after treatment with TNP can be attributed to the interaction of TNP with **HNT@Py**, and the average particle size of **HNT@Py** was able to detect TNP in water with high selectivity and sensitivity.^74^ The solid-state fluorescence spectra of **HNT@Py** in the presence and absence of TNP were measured at room temperature, as shown in Figure S8b. The fluorescence response of **HNT@Py** toward TNP was similar to the solution state in water, which indicated that the conformational properties of the modified nanoparticles do not change for solution and solid-state. These solid-state photophysical properties of **HNT@Py** led us to develop particle application kits for RGB analysis via **HNT@Py**-embedded nanofibers. The further investigation of the interaction of TNP with **HNT@Py** was conducted by FTIR and EDX analyses. The FTIR spectra of **HNT@Py** were recorded in the presence and absence of TNP, which demonstrated that the stretching vibration of the hydroxyl group is responsible for the additional peaks at 3544 and 3089 cm^–1^ in the FTIR spectrum of **HNT@Py** + TNP, as seen in Figure S9a. The peak at 3544 cm^–1^ indicated that TNP successfully forms hydrogen bonds with **HNT@Py**.^75^ Also, the peak at 3105 cm^–1^ for TNP shifted to 3089 cm^–1^ in **HNT@Py** + TNP, indicating hydrogen bonding with TNP.^76^ The data obtained from EDX and FTIR analysis showed that the -NH_2_ group of **HNT@Py** was prone to form hydrogen bonds with the phenolic oxygen of TNP.^77,78^ The structural properties of the **HNT@Py** and **HNT@Py** + TNP were also investigated by EDX analysis (Figure S9b,c). The EDX sample was prepared by combining **HNT@Py** with TNP. A solution of TNP in acetone was added to an aqueous dispersion of **HNT@Py**. The mixture was then subjected to rotary evaporation and vacuum drying, yielding a uniform solid sample. Considering the nitrogen, carbon, and oxygen atoms in the TNP structure, it was observed that after the interaction between the **HNT@Py** nanosensor and TNP, the percentages of C and N increased, while the percentages of Al and Si decreased as expected. The decrease in the oxygen percentage after interaction with TNP can be attributed to the replacement of oxygen-containing species on the surface by TNP and the hydrogen bonds formed between the oxygen atoms in the TNP structure and the surface. This observation aligns with the findings of the FTIR analysis of **HNT@Py** + TNP.^79,80^

**Figure 3:**
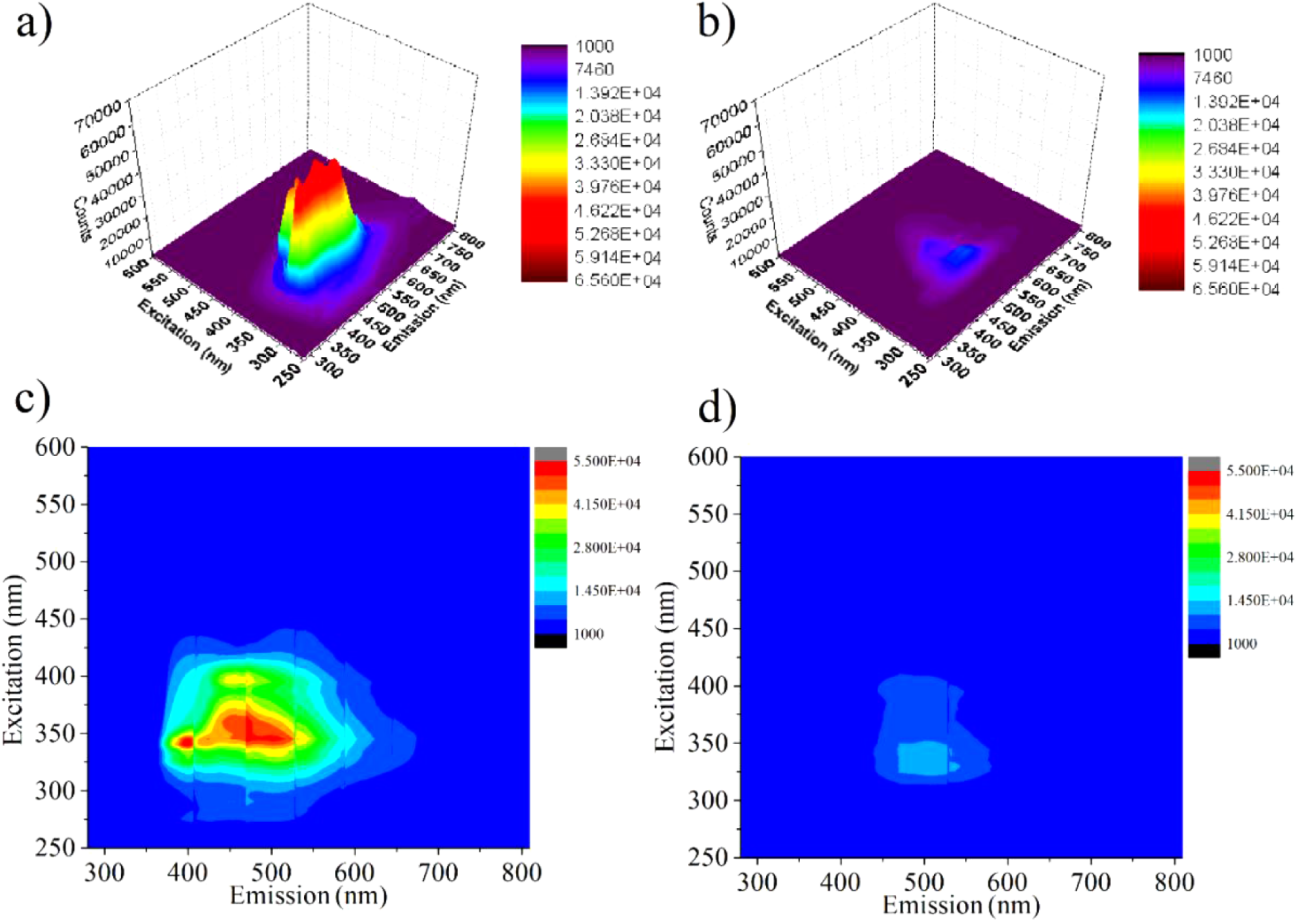
3D-emission spectra of (a) **HNT@Py**, (b) **HNT@Py** + TNP and EEM analyses of (c) **HNT@Py** and (d) **HNT@Py** + TNP in water.

The positions of emission band peaks, along with any observed blue or red shifts, provide important insights into the structure of the sensor system and analyte, encompassing aspects such as conformational changes, interactions, and heterogeneity. Consequently, three-dimensional (3-D) fluorescence and EEM analyses are frequently utilized in dynamic processes, especially for fluorimetric sensing.^81^ To evaluate the sensing mechanism and interactions, EEM analysis and 3D-fluorescence spectra of **HNT@Py** and **HNT@Py** + TNP were recorded (Figure 3). The 3D fluorescence graph of the presented sensor system showed that the dimeric emission of **HNT@Py**, which originated from pyrene, was situated in the range of 275–800 nm (Figure 3a). This emission was suppressed entirely when treated with TNP, without any shift toward blue or red (Figure 3b). Furthermore, the addition of TNP significantly altered the counterplot of **HNT@Py** (Figure 3c,d).

**Scheme 2:**
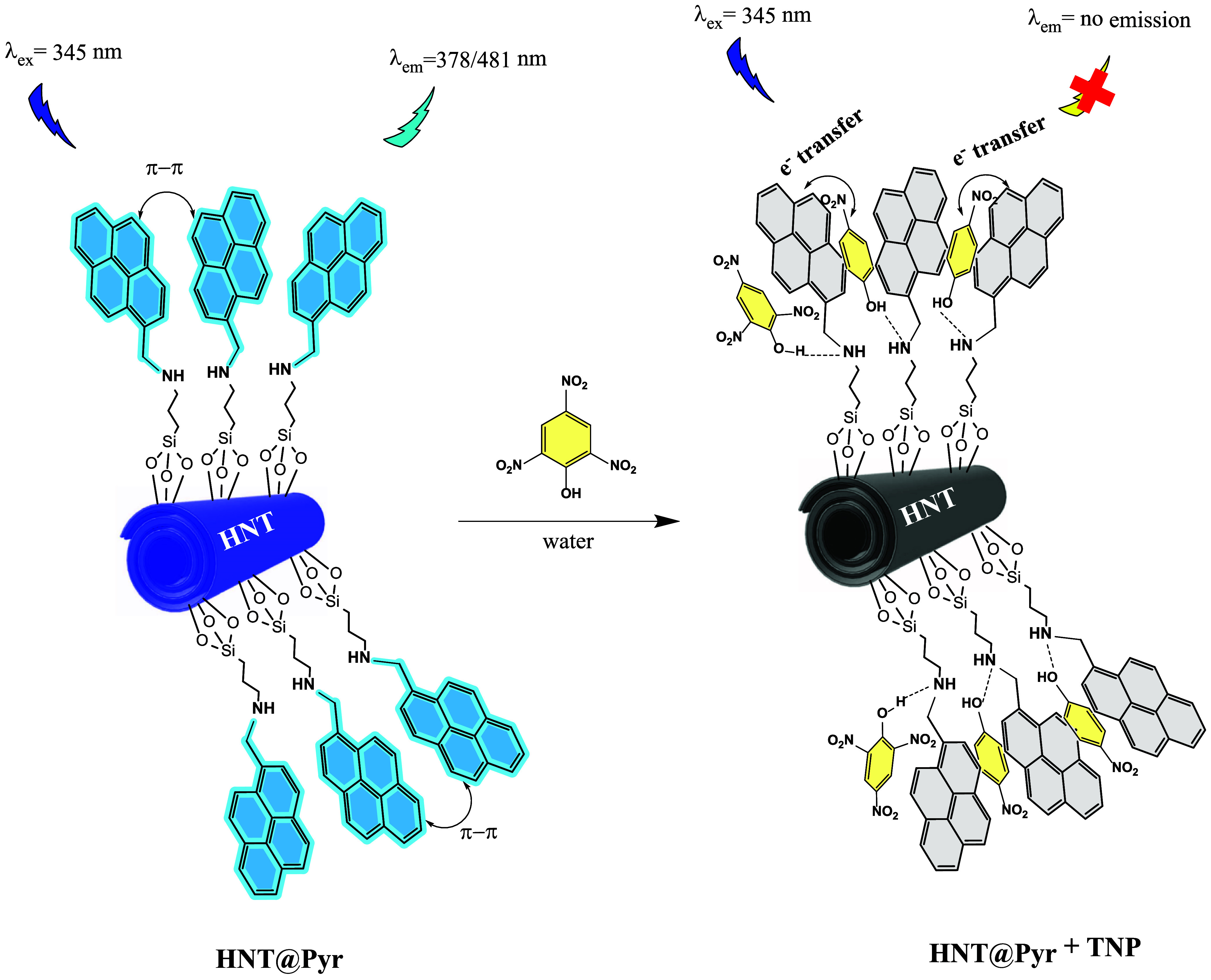
Proposed “Turn-Off” Signal Change and Interaction of **HNT@Py** with TNP

Depending on the interaction results, the fluorescence signal of the aqueous dispersion of bare **HNT@Py** experienced a significant decrease in the *I*_378_/*I*_481_ ratio, exhibiting a “turn-off” fluorescence response following the selective interaction with TNP. This effect is attributed to the photoinduced electron transfer (PET) mechanism occurring between the electron-deficient TNP and the electron-rich pyrene components of **HNT@Py**, which aligns with findings from fluorescence quantum yield measurements and spectroscopic analyses. Subsequently, the obtained “turn on–off” fluorescence response of **HNT@Py** could be simply employed for further TNP quenching signal detection. The suggested TNP sensing system is shown in Scheme 2.

### Analytical Performance of HNT@Py

**Figure 4:**
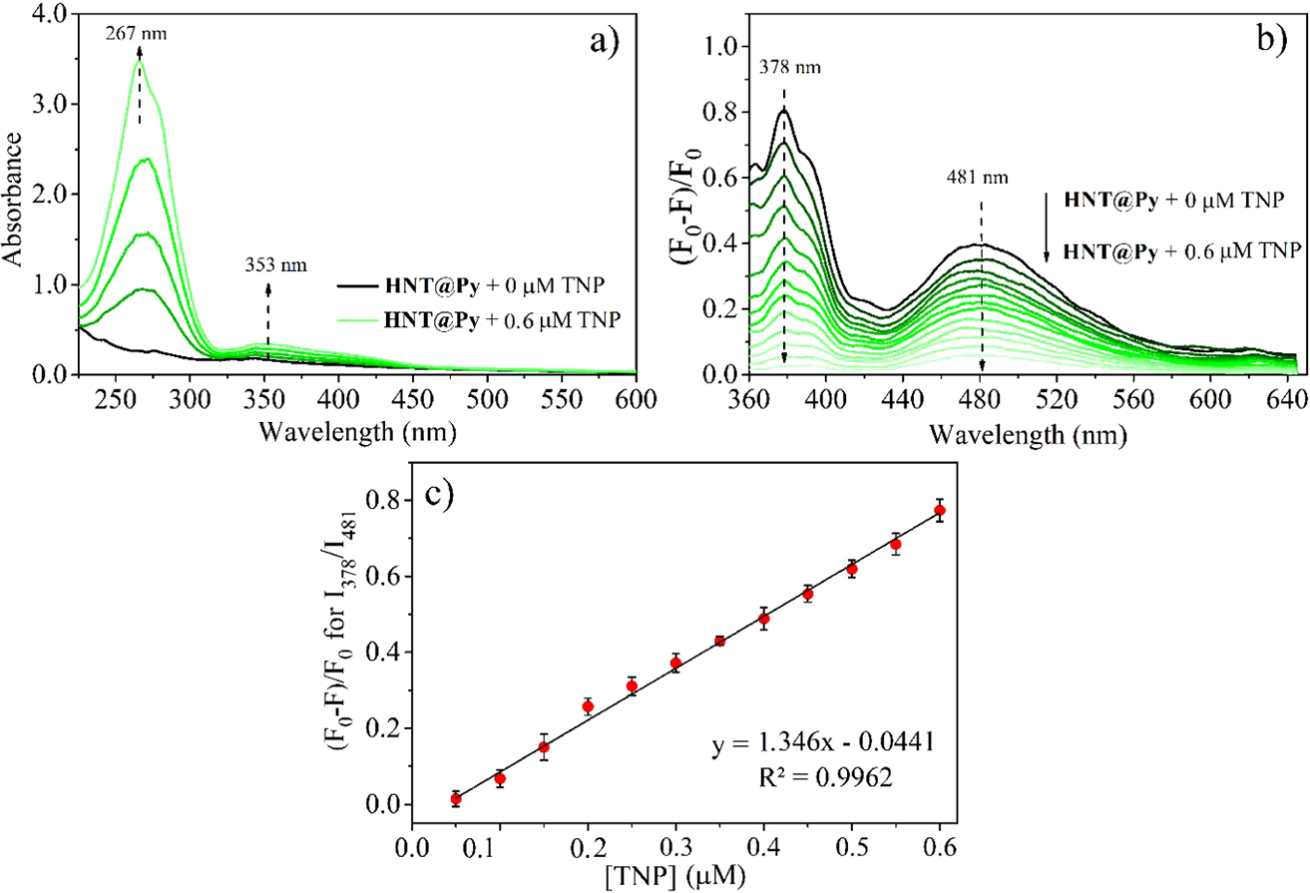
(a) Absorption and (b) fluorescence response change of **HNT@Py** after treatment with the gradual addition of TNP and (c) linear relative fluorescence response change of **HNT@Py** toward TNP in water.

The **HNT@Py** fluorescence nanosensor was used in real water, food, and soil samples to detect fluorometric TNP. This was accomplished by performing absorbance and fluorescence titration studies of **HNT@Py** under optimal conditions, gradually increasing the concentration of TNP (Figure 4). The UV–vis and fluorescence responses of **HNT@Py** toward increasing amounts of TNP were investigated under optimized conditions. The interaction between **HNT@Py** and TNP resulted in an increase in absorption at approximately 267 and 353 nm (Figure 4a), which is consistent with previous reports.^82^ The fluorescence signal of the **HNT@Py** nanosensor at 378 and 481 nm (λ_ex_= 345 nm) showed a linear turn on–off response change up to 0.60 μM (Figure 4b). The relative fluorescence response change of the **HNT@Py** nanosensor at *I*_378_/*I*_481_ was linear between 0.04 and 0.60 μM with the linear regression formula of *y* = 1.346[TNP] – 0.0441 (*R*^2^ = 0.9962), where *y* represents the relative fluorescence response change ratio at *I*_378_/*I*_481_ ([*F* – *F*_0_/*F*_0_]) of the **HNT@Py** nanosensor after the addition of TNP and [TNP] is the concentration of TNP at micromolar levels. Therefore, the linear correlation obtained between the TNP concentration and the relative fluorescence signal change of **HNT@Py** can serve as the calibration equation for the determination of TNP in real water, food, and soil samples.

The limits of detection (LOD) and quantification (LOQ) were established at 14.00 and 42.00 nmol·L^–1^, respectively, using the formulas 3σ/*K* and 9σ/*K* (where σ represents the standard deviation of the blank sample and *K* denotes the slope of the calibration curve). Furthermore, the precision of the fluorimetric determination method for TNP based on **HNT@Py** was evaluated with ten repeated analyses conducted under optimized conditions and given as the relative standard deviation (RSD%). The calculated RSD% for TNP was found to be 3.52%, indicating a high level of repeatability for the proposed determination method. The analytical parameters for TNP, derived from the relative fluorescence signal change of **HNT@Py**, are detailed in Table S2.

### Fluorometric Determination of TNP in Real Samples and Validation of the Assay

TNP is an anionic nitrophenol derivative that is highly mobile in soils and very water-soluble due to the hydroxyl group and three electron-withdrawing nitro groups.^83^ The water, food, and soil samples were used in real sample studies due to the high solubility in water and high mobility in the soil of TNP. Following the addition of TNP to the samples at different concentrations, the relative fluorescence responses of **HNT@Py** were determined following the procedure (*N* = 3) under optimal conditions. After that, the linear regression of the calibration graph was used to determine the recoveries of TNP concentrations. The results of the spike/recovery test, which restored the TNP concentration in line with the spiked concentration (95.52%–100.28%), are displayed in Table 2. Furthermore, after the spike and recovery tests were conducted, HPLC analysis was employed as a reference technique to evaluate the accuracy of the proposed determination method. The quantitative analysis of TNP using the HPLC method involved calibration curves generated by plotting TNP concentrations against the peak areas of the chromatograms (Figure S10). As tabulated in Table 2, the **HNT@Py** nanosensor-based fluorometric method agreed well with HPLC analysis. The statistical analysis of the results obtained from the proposed detection method (**HNT@Py**) was conducted to evaluate its performance. HPLC was applied by Student’s *t*-test, which pointed out the high accuracy of the **HNT@Py** sensor at the confidence level of 95% (Table S3).

**Table 2:** Determination of TNP in Soil, Wastewater and Apple Samples with **HNT@Py** and HPLC Analyses of TNP

		HNT@Py		HPLC	
Real Samples	Spiked (μmol ·L^–1^)	Found (μmol ·L^–1^)	Recovery (%)	Found (μmol ·L^–1^)	Recovery (%)
**Wastewater**	0	-		-	-
	30.00	29.04 ± 0.74	96.80	28.57 ± 0.52	95.23
	50.00	47.76 ± 1.22	95.52	48.37 ± 1.05	96.74
**Apple**	0	16.97 ± 0.25		17.57 ± 0.29	-
	30.00	45.43 ± 0.95	94.86	46.82 ± 0.75	97.50
	50.00	66.34 ± 1.40	98.74	66.86 ± 1.99	98.58
**Soil**	0	-		-	-
	30.00	28.68 ± 0.69	100.28	29.35 ± 0.48	97.83
	50.00	50.14 ± 1.25	95.60	48.73 ± 0.89	97.46

### RGB-Based Practical Application of TNP Detection via HNT@Py-Doped Electrospun Nanofibers

**Scheme 3:**
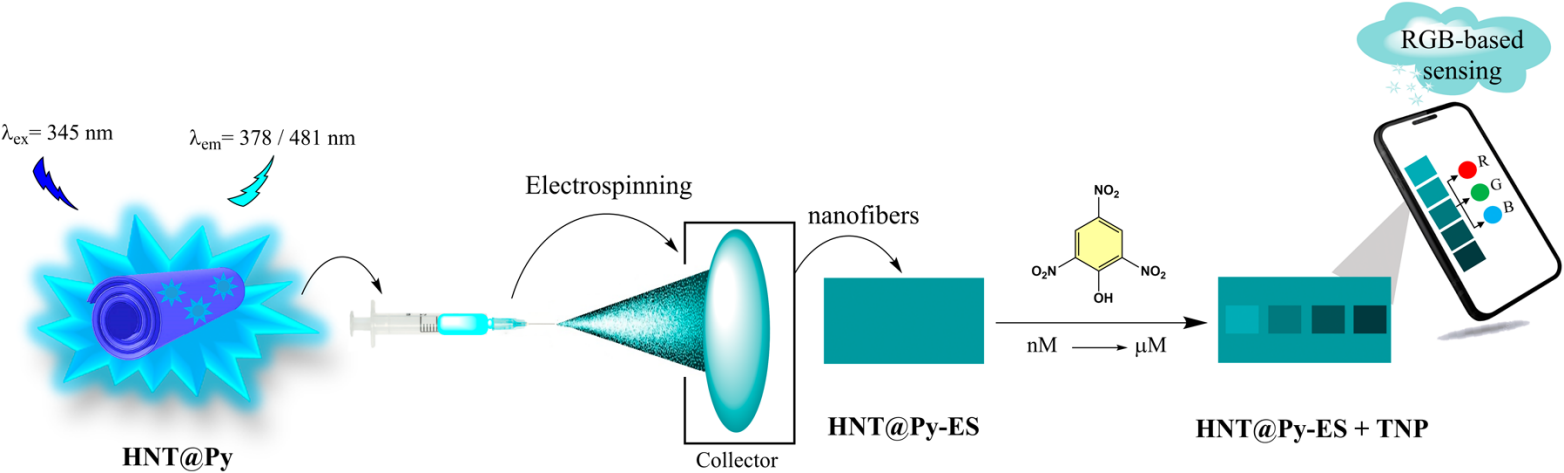
Illustration of **HNT@Py**-embedded Nanofiber Production (**HNT@Py-ES**) and Practical Application in the Solid State via RGB-Based Sensing of TNP Via **HNT@Py-ES**

PCL is recognized among electrospinnable polymers for its ability to create nanofibers with outstanding characteristics and composites containing different fluorescent dyes across a broad spectrum of compositions, resulting in light emission/quenching in response to specific stimuli.^84^ For this reason, **HNT@Py** nanostructures were dispersed into the PCL matrix using the electrospinning method to obtain regular morphology nanofibers (**HNT@Py-ES**). They were used for TNP detection with the fluorescence “turn on–off” method. The illustration of **HNT@Py**-embedded nanofiber fabrication (**HNT@Py-ES**) and its practical application in the solid state via RGB-based sensing of TNP with **HNT@Py-ES** are shown in Scheme 3.

**Figure 5:**
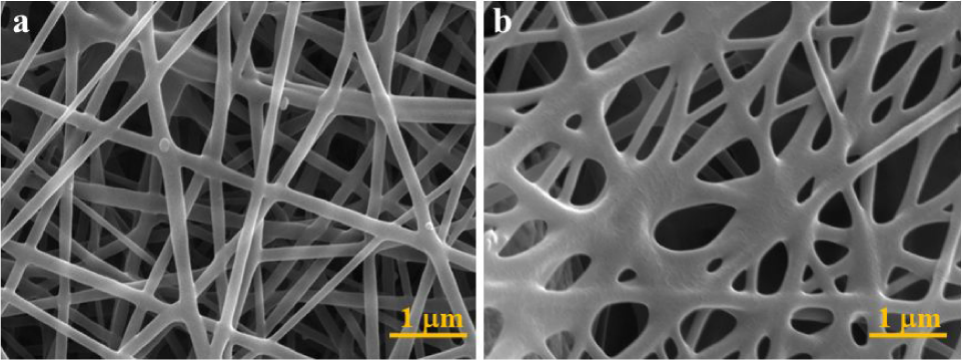
SEM images of nanofibers (a) PCL and (b) **HNT@Py**-embedded-PCL (**HNT@Py-ES**).

The SEM analysis was applied for the investigation of the membranes’ morphology to characterize their nanofiber nature (Figure 5). The PCL and **HNT@Py**-embedded-PCL (**HNT@Py-ES**) nanofibers showed fiber characteristics with individual fiber diameters of 165.11 ± 31.98 nm and 177.75 ± 47.30 nm, respectively. According to SEM images, the addition of **HNT@Py** increases the diameter of the nanofibers, which were determined to be relatively homogeneous and smooth.^85^

**Figure 6:**
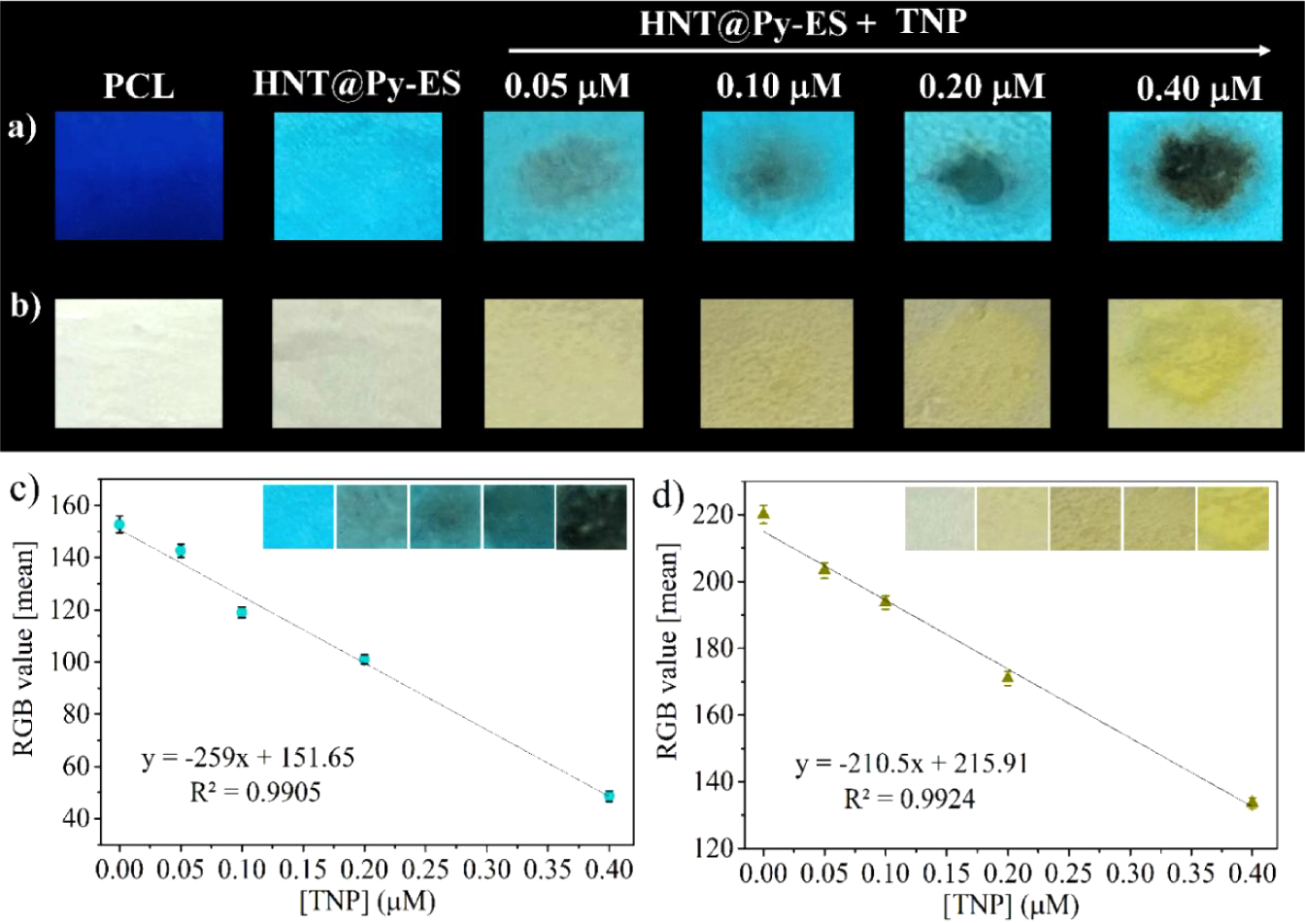
Color changes of **HNT@Py**-embedded-PCL (**HNT@Py-ES**) after addition of TNP under (a) UV light and (b) daylight. The obtained calibration curves after smartphone analysis of RGB values for **HNT@Py**-embedded-PCL (**HNT@Py-ES**) after addition of TNP under (c) UV light and (d) daylight.

The color signals from the modified nanofibers **HNT@Py-ES** were transformed into distinct red (R), green (G), and blue (B) values by utilizing a smartphone image in combination with the “Colorimeter” application for Android OS, specifically on a Samsung Galaxy A34 smartphone. The application established a linear correlation between the RGB values and TNP concentration in the range of 0.05 to 0.40 μM (Figure 6). Pure PCL does not exhibit fluorescence properties,^86^ but **HNT@Py** has intense bright blue emission. In Figure 6a, the bright blue color of the **HNT@Py**-embedded-PCL (**HNT@Py-ES**) nanofiber turned clearly brown/black under UV light with the increasing concentration of TNP. Also, the color of the **HNT@Py-ES** nanofiber in daylight changed from colorless to yellow (Figure 6b). The analysis of colorimetric changes in **HNT@Py-ES** nanofibers following treatment with TNP was conducted using RGB values to generate the calibration curves. These curves illustrated a strong linear correlation among the R, G, and B values and TNP concentrations up to 0.40 μM (Figure 6c,d). The findings indicate that the developed paper kits are suitable for rapid, accurate, and sensitive analysis of TNP in field applications.

The analytical performance of the **HNT@Py**-based fluorescent detection method for TNP determination was compared with that in previous reports (Table S4). While certain sensing systems have demonstrated a wider linear working range than **HNT@Py**, the presented system exhibited superior limits of detection (LOD) and selectivity compared to these previously reported systems. In addition, the use of **HNT@Py** in fully aqueous media to determine TNP is seen as superior compared with applications in organic or mixed aqueous media. Additionally, fluorescence quenching of **HNT@Py** was observed at lower TNP concentrations compared to studies in the literature.^87−89^ The comparison with previous reports demonstrated that pyrene-anchored halloysite nanotube (**HNT@Py**) can be easily used and is an important candidate as a fluorescence sensor for the rapid, effective, easy, and novel “turn-off” fluorescence measurements of TNP in real samples, detected with a low detection limit.

## Conclusions

In conclusion, a pyrene-anchored halloysite nanotube (**HNT@Py**) was used as a fluorescence nanosensor to propose new, easy, efficient, and quick “turn on–off” quantification of TNP in wastewater, apple, and soil. The thermal, morphological, and structural characterization of **HNT@Py** was carried out using fluorescence, UV–vis, XRD, TGA, FTIR, TEM, and SEM techniques. The steady-state fluorescence and absorption spectra were used to assess the optical behavior of **HNT@Py**. A specific “turn on–off” fluorescence reaction was observed for the fluorescence sensor capabilities of **HNT@Py** when it was exposed to TNP ions among various competitive species in fully aqueous media. The detection method showed a notable decrease in fluorescence when comparing the intensity ratio of monomer vs excimer (*I*_378_/*I*_481_) in water dispersion, after the selective interaction of **HNT@Py** with TNP. The present fluorescence sensor was employed to quantify TNP in various matrices, including soil, food, and wastewater samples. Validation of the methodology through spike/recovery tests and HPLC analysis demonstrated a high level of accuracy in measuring the TNP fluorescence sensor. The current fluorescence method demonstrated a detection limit of 14.00 nmol L^–1^ for TNP, with a linear working range from 0.04 to 0.60 μmol·L^–1^. Through interaction and analytical performance evaluation, it can be concluded that the fluorescence signal change of **HNT@Py** could be utilized for the detection of TNP using the PET mechanism between electron-deficient TNP and electron-rich **HNT@Py**. In addition, a system based on the RGB response of **HNT@Py**-embedded-PCL (**HNT@Py-ES**) nanofibers was developed for efficient, rapid, and real-time detection of TNP. These results indicate that **HNT@Py** and its facilitated nanofiber derivative, **HNT@Py-ES**, have the potential to serve as a straightforward and effective analytical tool for the selective and sensitive fluorescence detection of TNP in soil, food, and water samples through RGB analysis by a smartphone application.

## Supporting Information Available

The characterization spectra of **HNT@Py** , some photophysical measurements, and HPLC analysis (PDF)
