# Supplementary material for: Highly Selective Pyrene-Anchored Halloysite Nanotube for Fluorometric Determination of 2,4,6-Trinitrophenol in Environmental and Food Samples
Source: ACS Omega. 2025 Feb 18;10(8):7949–63. doi: 10.1021/acsomega.4c08857 (PMC11886659; doi:10.1021/acsomega.4c08857)
Supplement: Supplementary file 1 — ao4c08857_si_001.pdf [file ao4c08857_si_001.pdf]

Supplementary Information

Highly Selective Pyrene Anchored Halloysite Nanotube for Fluorometric Determination of 2,4,6-trinitrophenol in Environmental and Food Samples

Vildan Sanko[a,b,c], İpek Ömeroğlu[a], Ahmet Şenocak[a], Süreyya Oğuz Tümay[a,d,e]*

[a]Department of Chemistry, Gebze Technical University, Kocaeli 41400, Türkiye

[b]Department of Chemistry, Hacettepe University, Ankara 06800, Türkiye

[c]METU MEMS Center, Ankara 06530, Türkiye

[d]Department of Chemistry, Atatürk University, Erzurum 25240, Türkiye

[e]Department of Nanoscience and Nanoengineering, Atatürk University, Erzurum 25240, Türkiye

* Corresponding author:

Dr. Süreyya Oğuz TÜMAY

orcid.org/0000-0002-0453-4021

e-mail: sotumay@atauni.edu.tr

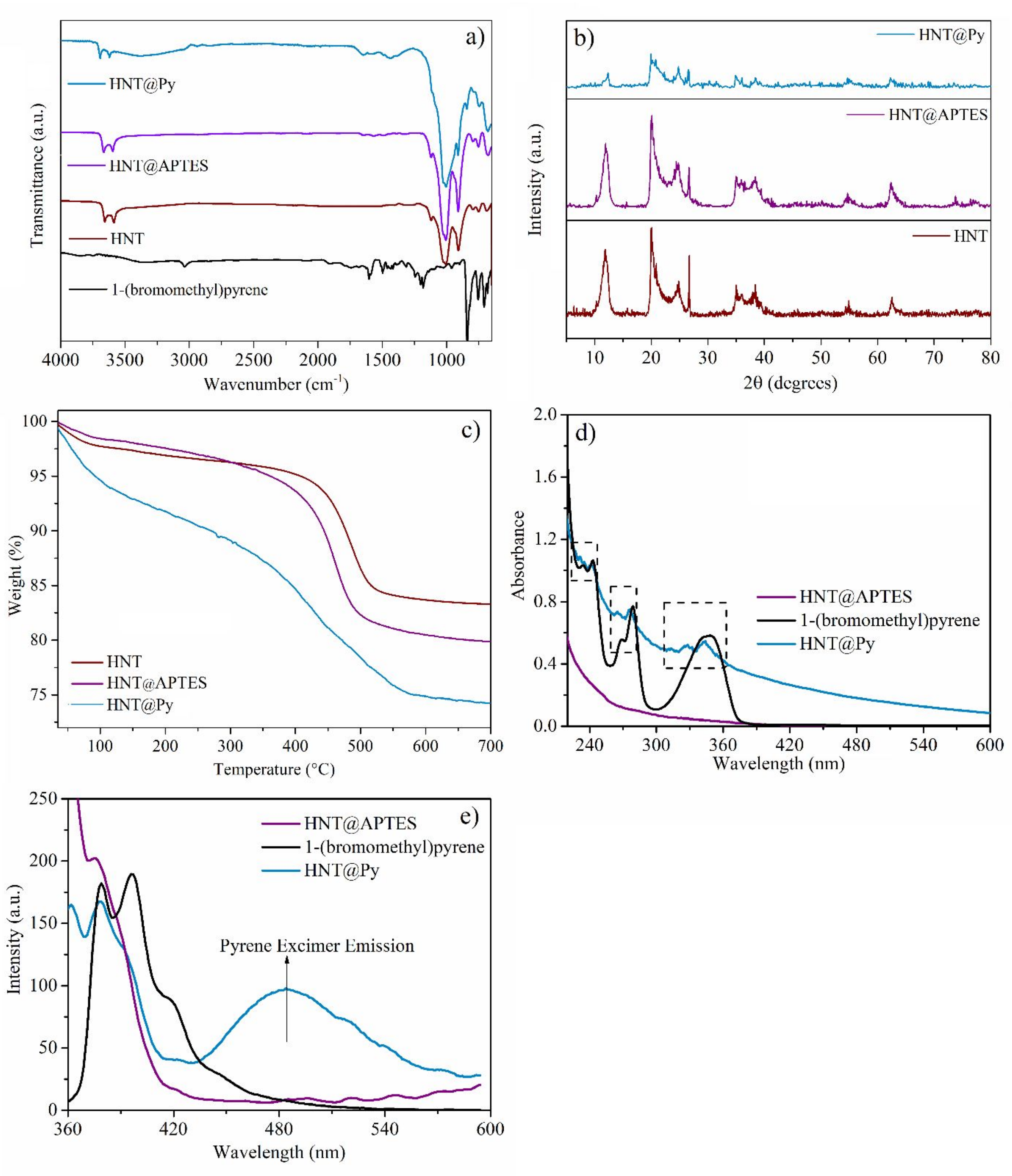

Fig. S1. The characterization results of **HNT@Py**; a) FTIR, **b)** XRD, **c)** TGA, **d**) UV-Vis and **e**) fluorescence spectra.

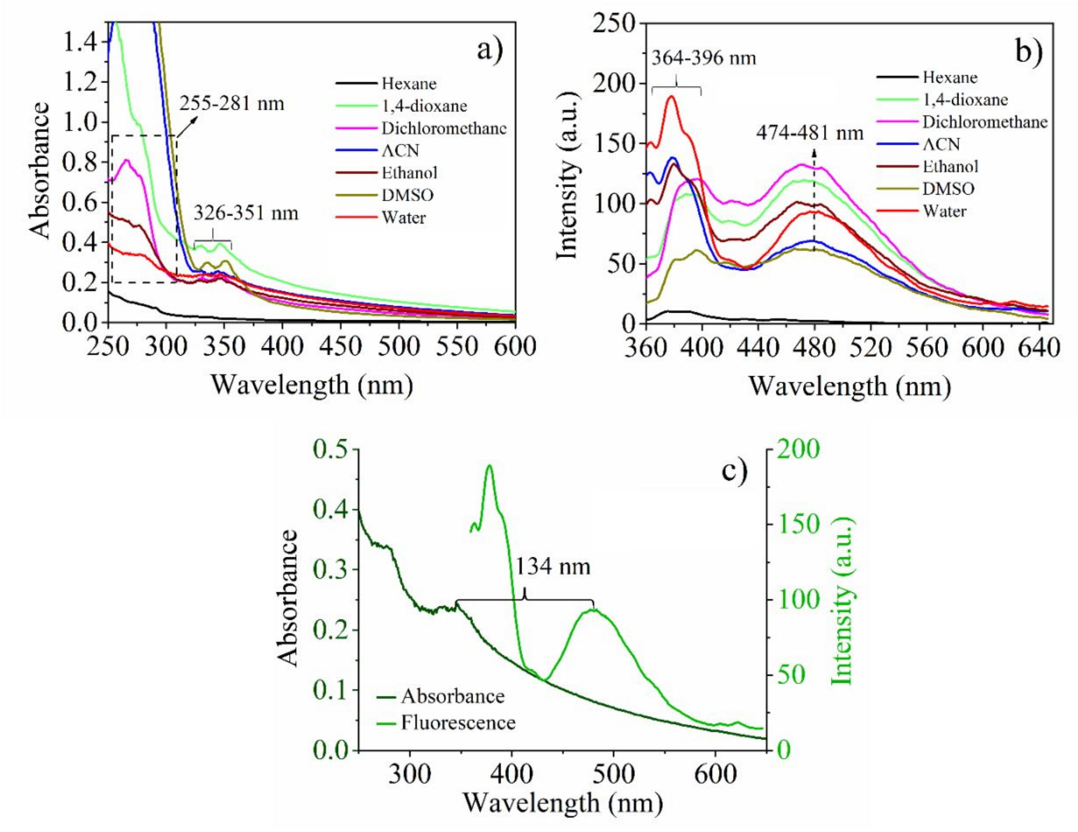

Fig. S2. **a)** UV-Vis absorption, **b)** fluorescence spectra (λ_{ex}= 345 nm) of 0.50 mg.mL^{-1} of **HNT@Py** in different mediums, and **c)** Stokes Shift in water.

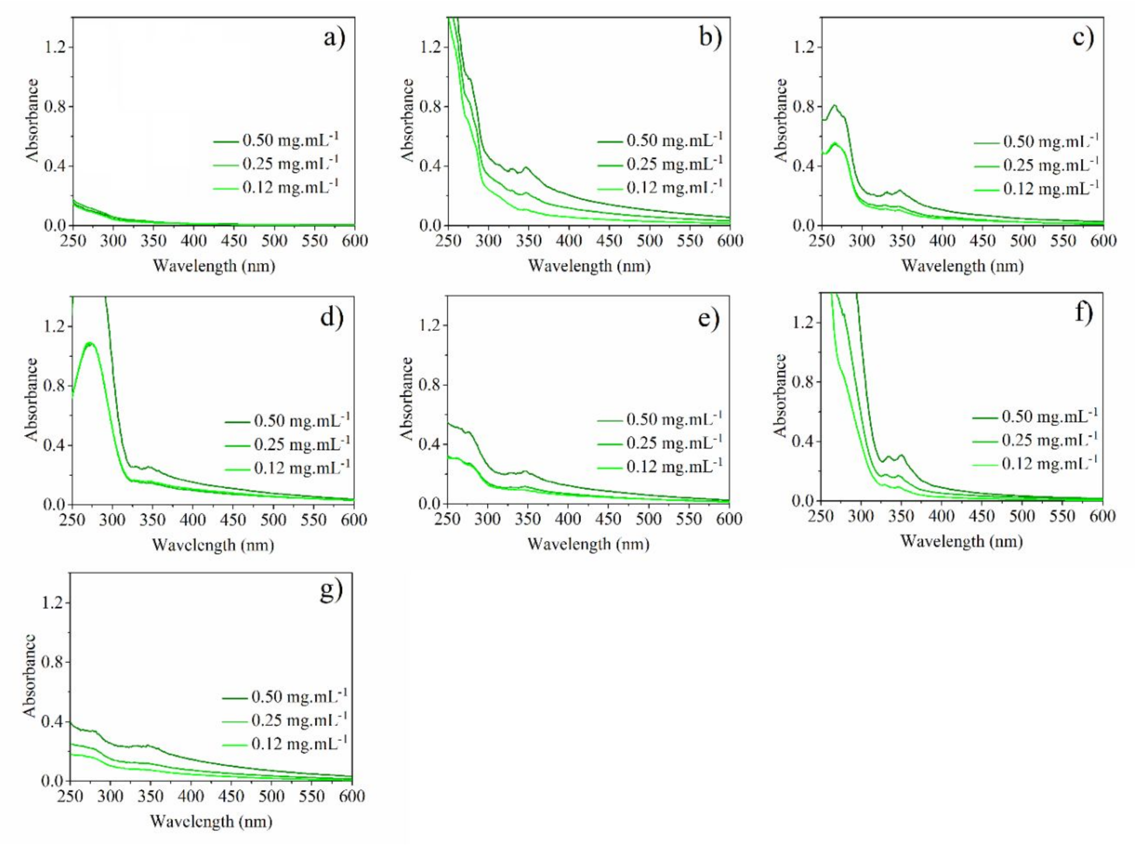

Fig. S3. The UV-Vis spectra for different concentration of **HNT@Py** in various solvents **a)** n-hexane, **b**) 1,4-dioxane, **c**) dichloromethane, **d**) ACN, **e**) EtOH, **f**) DMSO and **g**) water.

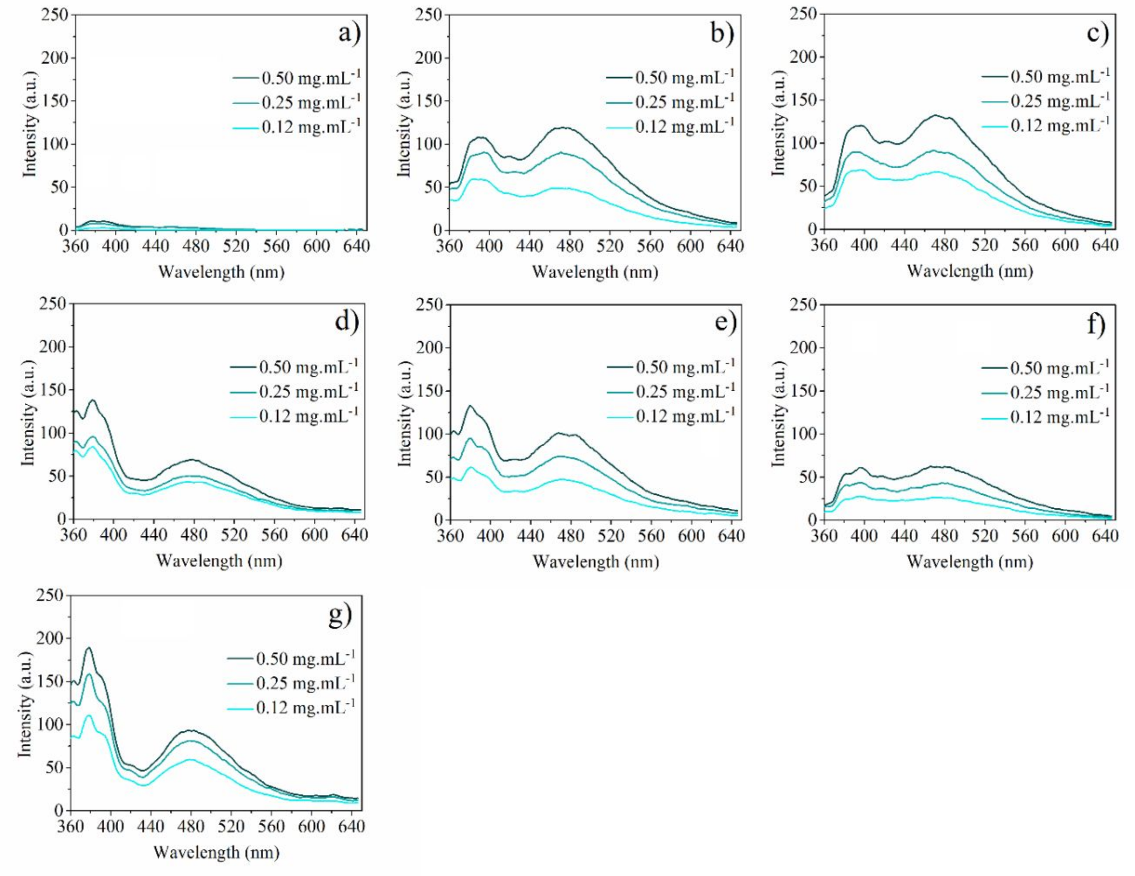

Fig. S4. The fluorescence spectra for different concentration of **HNT@Py** in various solvents **a)** n-hexane, **b**) 1,4-dioxane, **c**) dichloromethane, **d**) ACN, **e**) EtOH, **f**) DMSO and **g**) water.

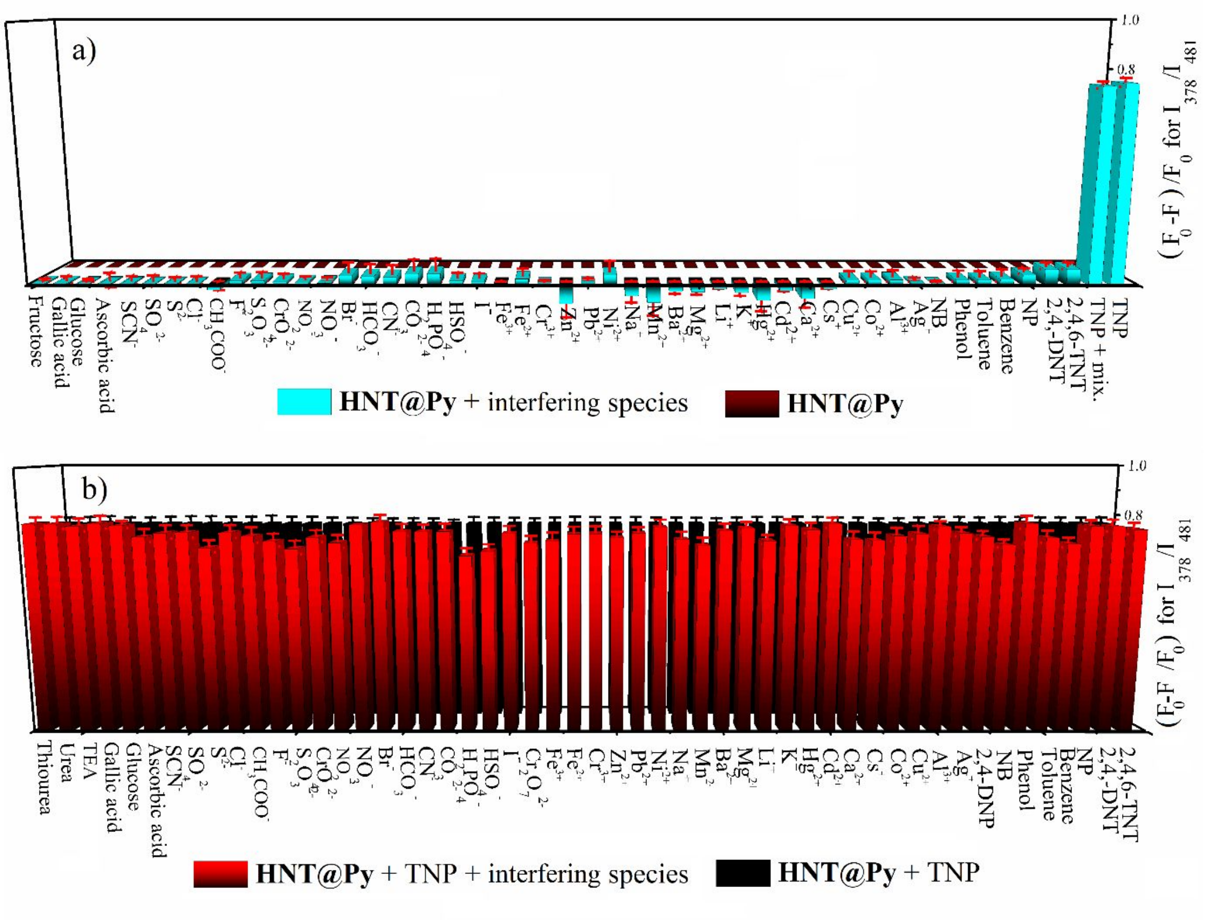

Fig. S5. Relatively changes of **a) HNT@Py** fluorescence signal and **b) HNT@Py** + TNP fluorescence signal in water after adding various interfering species.

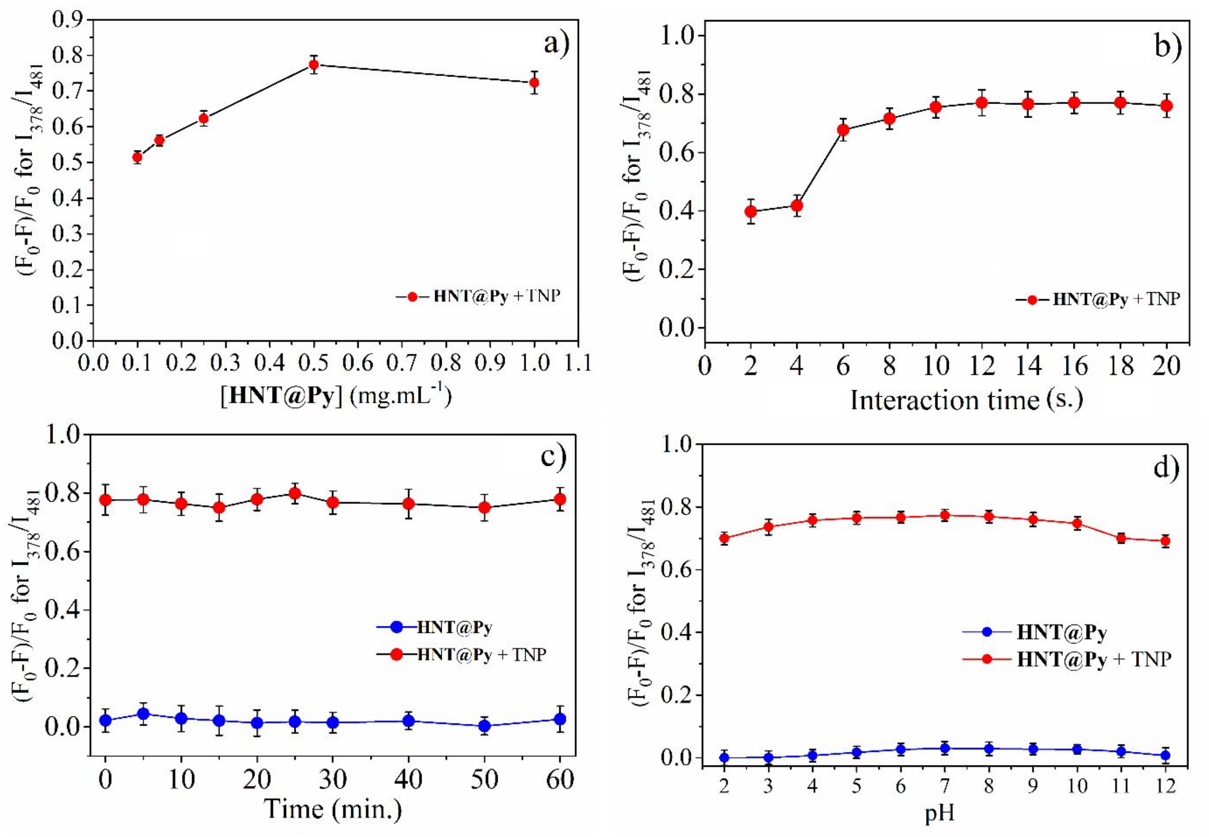

Fig. S6. The relative fluorescence responses of **HNT@Py** and **HNT@Py** + TNP in water at different **a)** initial sensor concentration, **b)** interaction time, **c)** times of light exposure and **d)** pH.

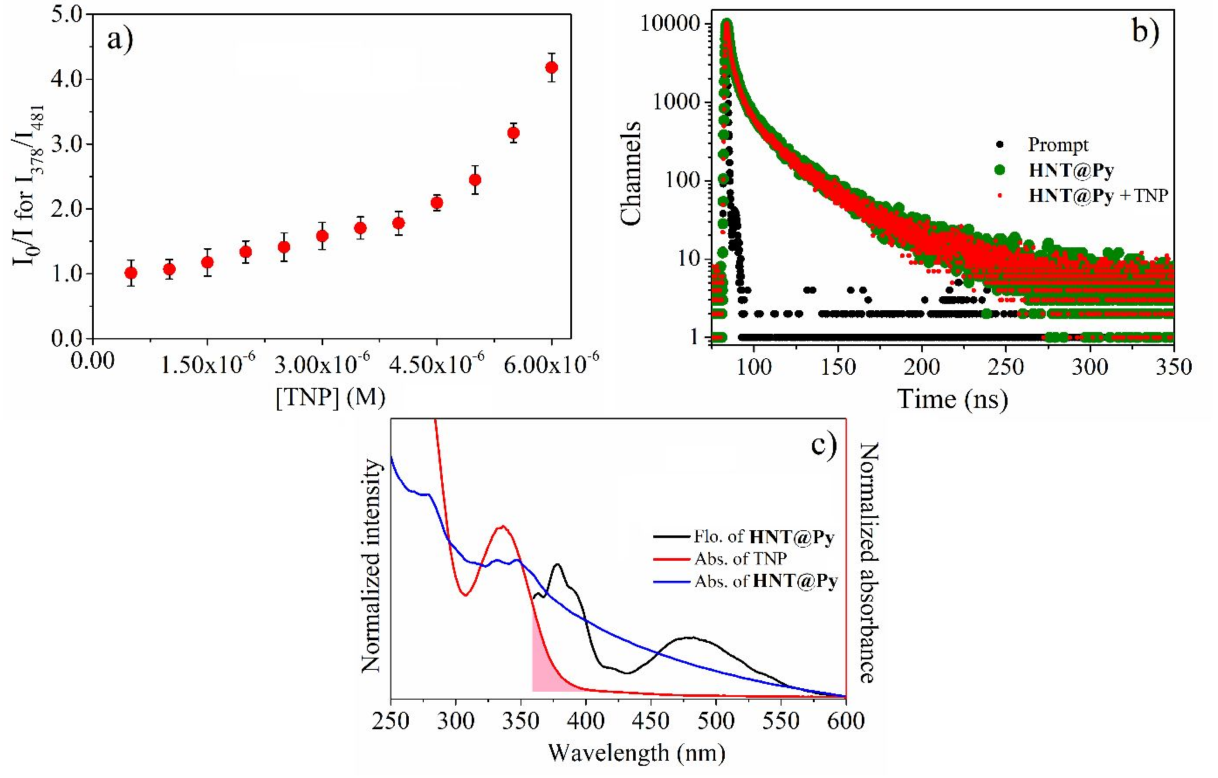

Fig. S7. **a)** Stern-Volmer graph, **b)** fluorescence lifetime analyses for **HNT@Py** and **HNT@Py** + TNP in water and **c)** UV-Vis absorption and fluorescence response of TNP and **HNT@Py**.

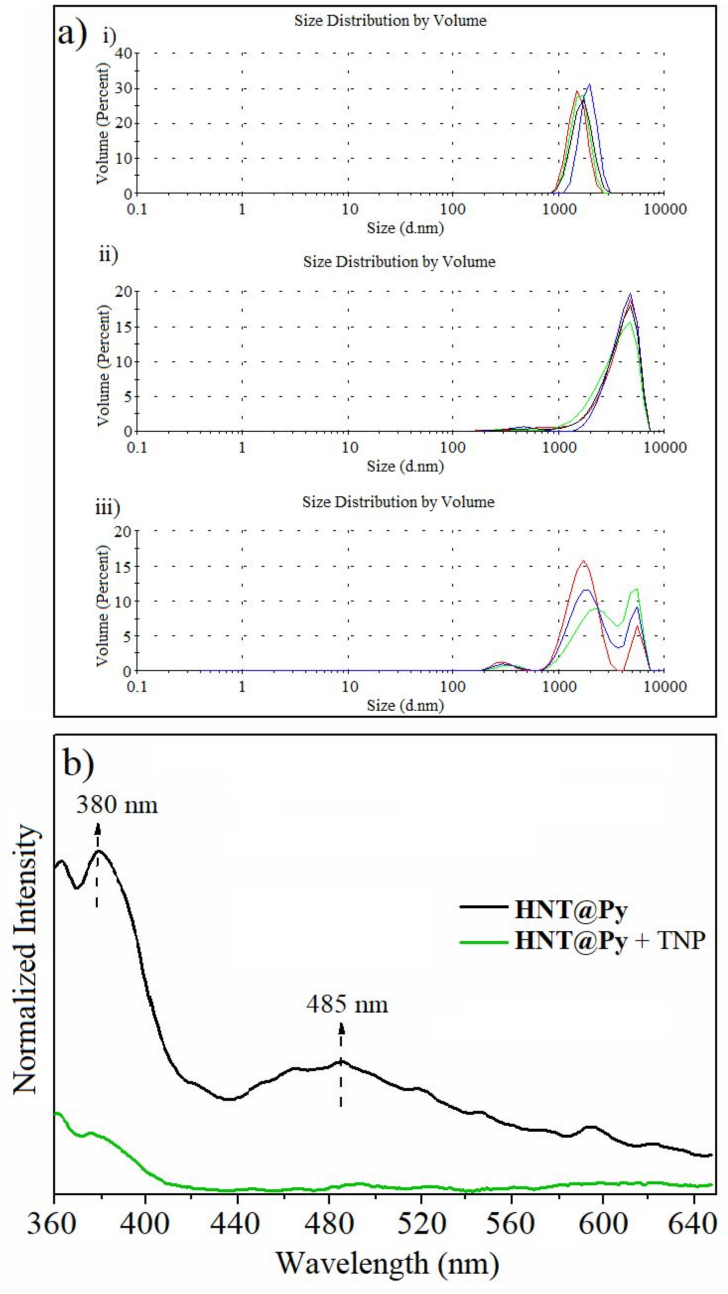

Fig. S8. **a)** Particle-size: **i) HNT**, **ii) HNT@Py**, **iii) HNT@Py** + TNP and **b)** solid-state fluorescence analyses of **HNT@Py** and **HNT@Py +** TNP.

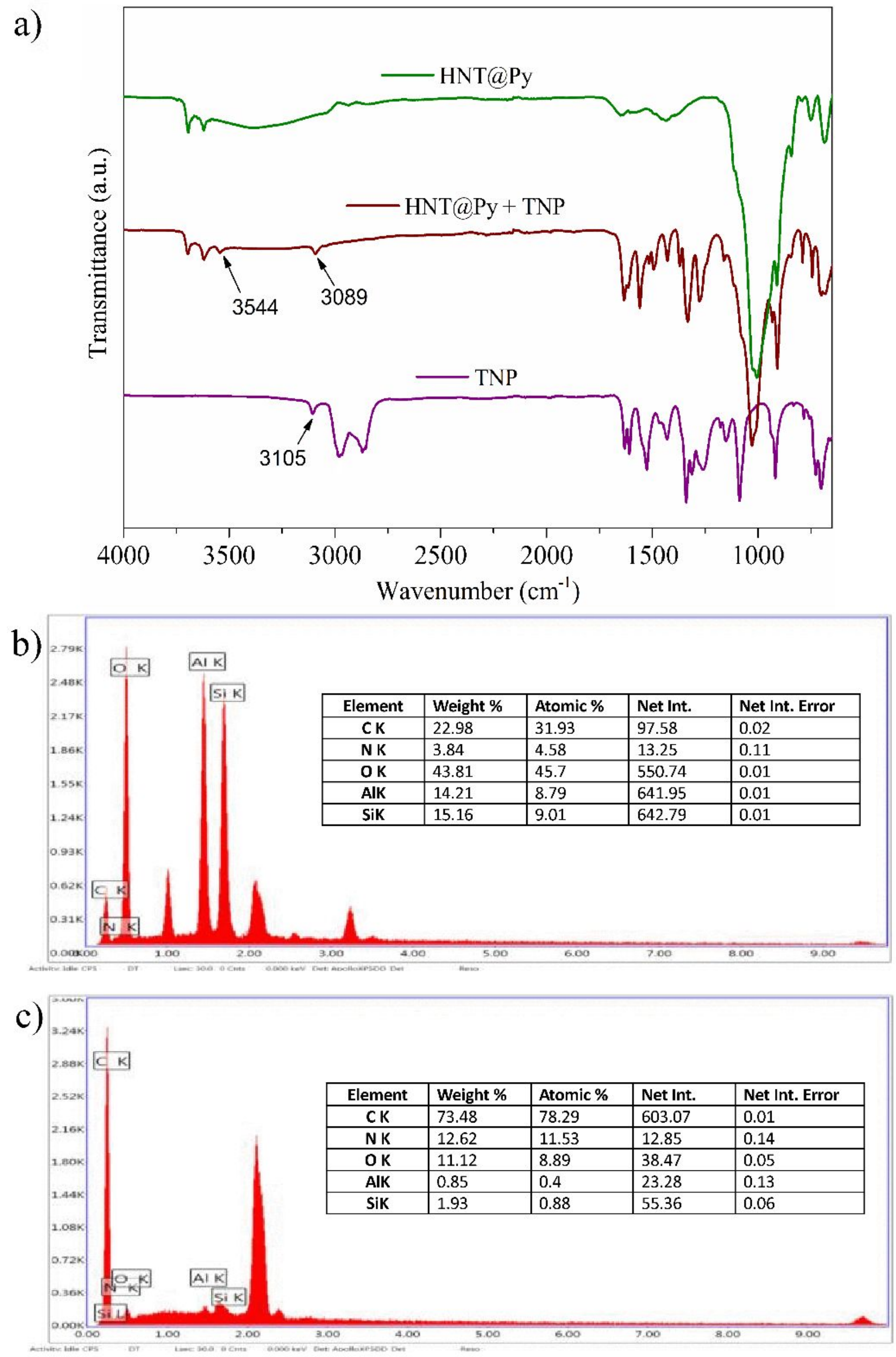

Element	Weight %	Atomic %	Net Int.	Net Int. Error
C K	22.98	31.93	97.58	0.02
N K	3.84	4.58	13.25	0.11
O K	43.81	45.7	550.74	0.01
AlK	14.21	8.79	641.95	0.01
SiK	15.16	9.01	642.79	0.01

Element	Weight %	Atomic %	Net Int.	Net Int. Error
C K	73.48	78.29	603.07	0.01
N K	12.62	11.53	12.85	0.14
O K	11.12	8.89	38.47	0.05
AlK	0.85	0.4	23.28	0.13
SiK	1.93	0.88	55.36	0.06

Fig. S9. FTIR (**a**) and EDX spectra of **HNT@Py** (**b**), **HNT@Py** + TNP (**c**)

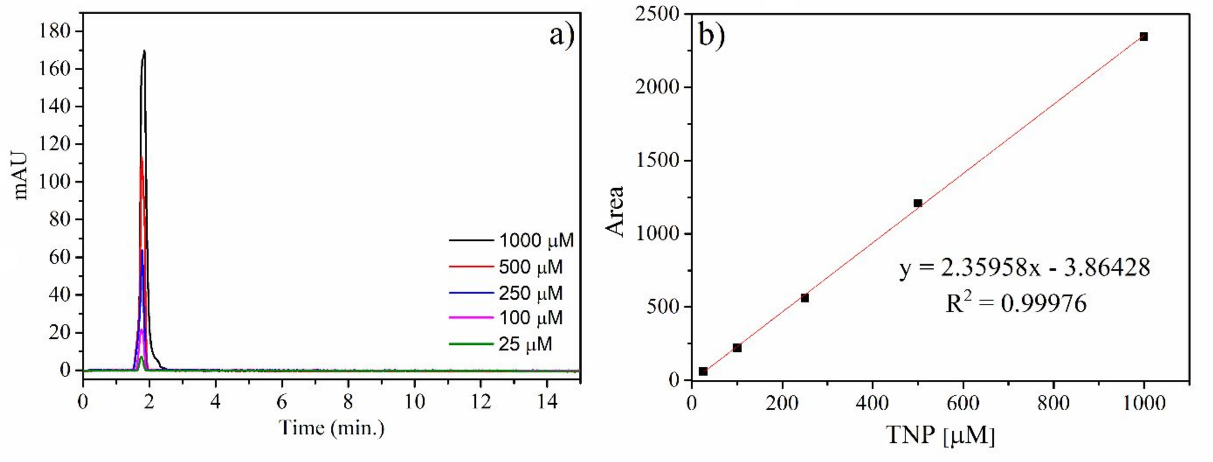

Fig. S10. a) the HPLC chromatogram of various concentration of TNP and **b)** calibration curve of TNP.

Table S1: The photophysical calculations for **HNT@Py**.

			ε (L g^{-1} cm^{-1}) x10^3								
	λ_{abs} (nm)	λ_{em} (nm)	Hxn	THF	DCM	ACN	EtOH	DMSO	Su	Φ_F	τ_0 (ns)
**HNT@Py**	267/353	378/481	0.053	0.71	0.426	0.494	0.408	0.57	0.466	0.20	25.48

Table S2: The analytical parameters for determination of TNP with **HNT@Py**.

**Parameter**	**HNT@Py**
$\lambda_{excitation}$ (nm)	345
$\lambda_{emission}$ (nm)	378/481
Time before measurement (s.)	10
Sensor concentration (mg.mL^{-1})	2.00
Final volume (mL)	2
Sensing medium	water
Linear working range (μmol.L^{-1})	0.04-0.60
Limit of detection (LOD) (nmol.L^{-1})	14.00
Limit of quantification (LOQ) (nmol.L^{-1})	42.00

Correlation coefficient (R^2)	0.9962
RSD%	3.52

Table S3. The statistical evaluation of the determination results of TNP with Student's t-test.

**TNP**	**s**	**X_R**	$\overline{X}$	$t_{exp.}=\frac{\lvert X_R-\overline{X}\rvert}{s/\sqrt{N}}$	$t_{ref.}$	**Results**
**Wastewater**	2.54	30.00	29.04	0.65	4.3	0.65<4.3
**Apple**	2.09	17.57	16.97	0.34	4.3	0.50<4.3
**Soil**	1.37	30.00	28.68	0.79	4.3	0.79<4.3

Table S4. The comparison of some reported fluorescence sensor techniques of TNP with **HNT@Py.**

**Materials**	**Real Sample**	**Analysis medium**	**Linear Range (µmol L^{-1})**	**LOD (nmol L^{-1})**	**Ref.**
Sulfur-doped GQDs	Pond water<br>Rainwater	PBS	0.1-100.0	9.3×10^{-5}	[1]
Pyrene-Functionalized Nanoporous COFs	-	THF	0-62.0	4.0×10^{-4}	[2]
Pyrene-PDA CHOFs	Tap water<br>Soil	Ethanol	0-200.0	2.14×10^{-3}	[3]
Thiazole linked COFs	Tap water<br>Drinking water	1,4-Dioxane	2.0-12.0	1.70×10^{-4}	[4]
Lysozyme-capped CdS QDs	Tap water<br>Spring water	Tris–HCl buffer	0.5-15.0	1.0×10^{-4}	[5]
Yellow-emissive carbon dots	River water<br>Tap water	Phosphate buffer	0.2-130.0	56	[6]
Photoluminescent carbon nanodots	Running water<br>Lake water	Water	0.1-100	28	[7]
**HNT@Py**	Wastewater<br>Apple<br>Soil	Water	0.04-0.60 µM	14.00	This work

References

(1) Kadian, S.; Manik, G. A highly sensitive and selective detection of picric acid using fluorescent sulfur-doped graphene quantum dots. *Luminescence* **2020**, *35* (5), 763-772. DOI: https://doi.org/10.1002/bio.3782 (acccessed 2024/08/13).
(2) Tan, X.; Yang, C.; Xie, Y.; Gou, Q.; Zhang, R.; Ao, E.; Zhou, X.; Chen, Z.; Wang, Q.; Fu, L. Pyrene-Functionalized Nanoporous Covalent Organic Frameworks with a Donor–Acceptor Property for 2,4,6-Trinitrophenol Detection. *ACS Applied Nano Materials* **2024**, *7* (14), 16789-16798. DOI: 10.1021/acsanm.4c02840.
(3) Huo, T.; Yang, B.; He, Y. New porous organic framework Py-PDA CHOF as fluorescent chemosensor for detecting 2,4,6-trinitrophenol in environmental samples. *Microchemical Journal* **2024**, *205*, 111271. DOI: https://doi.org/10.1016/j.microc.2024.111271.
(4) Ali, Z.; Huo, T.; Zhang, Y.; Wang, G. Thiazole linked covalent organic framework as fluorescent chemosensor for selective and sensitive detection of TNP. *Microchemical Journal* **2024**, *200*, 110340. DOI: https://doi.org/10.1016/j.microc.2024.110340.
(5) Na, W.; Liu, X.; Pang, S.; Su, X. Highly sensitive detection of 2,4,6-trinitrophenol (TNP) based on lysozyme capped CdS quantum dots. *RSC Advances* **2015**, *5* (63), 51428-51434, 10.1039/C5RA06101F. DOI: 10.1039/C5RA06101F.
(6) Zhang, S.; Wang, H.; Li, Y.; Data, F. Y.; Wang, Q.; Jiao, L. Bright-yellow-emissive carbon dots with a large Stokes shift for selective fluorescent detection of 2, 4, 6-trinitrophenol in environmental water samples. *Materials Letters* **2020**, *263*, 127208. DOI: https://doi.org/10.1016/j.matlet.2019.127208.
(7) Liu, M. L.; Chen, B. B.; Liu, Z. X.; Huang, C. Z. Highly selective and sensitive detection of 2,4,6-trinitrophenol by using newly developed blue–green photoluminescent carbon nanodots. *Talanta* **2016**, *161*, 875-880. DOI: https://doi.org/10.1016/j.talanta.2016.08.046.
